# CSF1R Inhibition Reduces Microglia Proliferation, Promotes Tissue Preservation and Improves Motor Recovery After Spinal Cord Injury

**DOI:** 10.3389/fncel.2018.00368

**Published:** 2018-10-16

**Authors:** Yannick Nicolas Gerber, Guillaume Patrick Saint-Martin, Claire Mathilde Bringuier, Sylvain Bartolami, Christophe Goze-Bac, Harun Najib Noristani, Florence Evelyne Perrin

**Affiliations:** ^1^University of Montpellier, Montpellier, France; ^2^INSERM, U1198, Montpellier, France; ^3^EPHE, Paris, France; ^4^UMR 5221 CNRS, University of Montpellier, Montpellier, France

**Keywords:** spinal cord injury, reduced microglia proliferation, colony stimulating factor 1 receptor, GW2580, motor recovery, reduced gliosis, reduced microcavity

## Abstract

Spinal cord injury (SCI) induces a pronounced neuroinflammation driven by activation and proliferation of resident microglia as well as infiltrating peripheral monocyte-derived macrophages. Depending on the time post-lesion, positive and detrimental influences of microglia/macrophages on axonal regeneration had been reported after SCI, raising the issue whether their modulation may represent an attractive therapeutic strategy. Colony-stimulating factor 1 (CSF1) regulates microglia/macrophages proliferation, differentiation and survival thus, pharmacological treatments using CSF1 receptor (CSF1R) inhibitors had been used to ablate microglia. We analyzed the effect of chronic (10 weeks) food diet containing GW2580 (a CSF1R inhibitor) in mice that underwent lateral spinal cord hemisection (HS) at vertebral thoracic level 9. Treatment started 4 weeks prior to SCI and continued until 6 weeks post-lesion. We first demonstrate that GW2580 treatment did not modify microglial response in non-injured spinal cords. Conversely, a strong decrease in proliferating microglia was observed following SCI. Second, we showed that GW2580 treatment improved some parameters of motor recovery in injured animals through better paw placement. Using *in* and *ex vivo* magnetic resonance imaging (MRI), we then established that GW2580 treatment had no effect on lesion extension and volume. However, histological analyses revealed that GW2580-treated animals had reduced gliosis and microcavity formation following SCI. In conclusion, CSF1R blockade using GW2580 specifically inhibits SCI-induced microglia/macrophages proliferation, reduces gliosis and microcavity formations and improves fine motor recovery after incomplete SCI. Preventing microglial proliferation may offer therapeutic approach to limit neuroinflammation, promote tissue preservation and motor recovery following SCI.

## Introduction

Spinal cord injuries (SCIs) affect between 2.5 million and 4 million patients worldwide (van den Berg et al., 2010). Depending on the level and extent of the injury, symptoms range from minimal sensory/motor deficits to complete tetraplegia. Mechanisms underlying the pathophysiology of SCI are divided into two broad successive phases: the primary phase involves the initial mechanical injury (Rowland et al., 2008) followed by a secondary phase comprising several degenerative processes such as ischemia, vascular disruption, oxidative stress, excitotoxicity, neuroinflammation, gliosis, tissue necrosis and microcavity formation that altogether orchestrate neuronal demise (Sekhon and Fehlings, 2001). There is no curative treatment for any symptoms associated with SCI. Adult neurons have the intrinsic capacity for re-growth after injury, however, regeneration does not occur spontaneously (David and Aguayo, 1981) due to a non-favorable environment surrounding the lesion site. Following SCI, a glial scar, mainly composed of astrocytes and microglia, surrounds the injury site and creates a major impediment to spontaneous axonal re-growth (Fawcett and Asher, 1999).

Microglia, the main immune cells of the central nervous system (CNS) are highly sensitive to homeostatic changes in their environment and are involved in the initial stages of neuroinflammatory response following trauma (Kettenmann et al., 2011). SCI elicits a robust and highly coordinated inflammatory response that includes the rapid activation and migration of microglia concomitant with the infiltration and recruitment of peripheral monocytes derived macrophages within the lesion site (for review see, David and Kroner, 2011). Microglia and monocytes are derived from myeloid cell lineage as reflected by their common expression of surface receptors and signaling molecules (Schmitz et al., 2009). Following SCI, microglia play both detrimental and beneficial roles through their contribution, on the one hand, to secondary damages associated with SCI, and on the other hand, their involvement in neuroprotection (for review see, David and Kroner, 2011). Specifically, activated microglia release several pro-inflammatory mediators such as nitric oxide and cytokines (IL-1β, TNF-α, IL-6) that participate in neuroinflammation and lesion expansion (for review see, David and Kroner, 2011). Moreover, microglia activation participates, in central neuropathic pain development after SCI (Walters, 2014). Although neuroinflammation after SCI is generally considered detrimental for axonal regeneration, several studies clearly demonstrate beneficial roles for subsets of macrophages in CNS debris removal (Perrin et al., 2005), axonal regeneration, and re-myelination as well as through the expression of neurotrophic factors (Lambertsen et al., 2009; Mukaino et al., 2010; Plemel et al., 2014; Wlodarczyk et al., 2015; Noristani et al., 2017b). Similarly, other studies report an anti-inflammatory role of monocytes derived macrophages that is mediated by interleukin 10 overexpression, after SCI (Popovich et al., 1999; Shechter et al., 2009). Altogether, these studies thus highlight both favorable and detrimental influences of microglia/macrophages in CNS pathophysiology.

Targeting key cellular actors of neuroinflammation (microglia/macrophages, lymphocytes or neutrophils) have been reported to improve neurological outcome following SCI (Hawthorne and Popovich, 2011; David et al., 2012). Therapeutic approaches using corticosteroids to block neuroinflammation and limit secondary injury had reached clinical trials in SCI, traumatic brain injury and stroke but have not yielded significant beneficial outcomes (Harlan and Winn, 2002; Rigg and Zafonte, 2006; del Zoppo, 2010).

The colony-stimulating factor 1 (CSF1) regulates, proliferation, differentiation and function of macrophage lineage cells through binding to its specific receptor (CSF1R; Patel and Player, 2009). Under physiological conditions, microglia are the only cells in the CNS that express CSF1R (Erblich et al., 2011) and CSF1R inhibitors have recently been used to suppress microglia reactivity in different conditions. CSF1R inhibition not only leads to microglial demise but also other CSF1R-expressing cells both *in vitro* and *in vivo* (Elmore et al., 2014). Moreover, CSF1R is involved in inflammation-mediated activation by macrophages (Pixley and Stanley, 2004; Hamilton et al., 2008; Hamilton, 2008). CSF1R inhibitors such as PLX3397, BLZ945 and GW2580 have been administered orally to manipulate microglia in the CNS (for review see, Wieghofer et al., 2015). Selective inhibition of CSF1R using PLX3397 transiently eliminates ~99% of microglia in the adult mouse brain without behavioral or cognitive impairment (Elmore et al., 2014). New microglia repopulate the brain within 1 week of inhibitor delivery cessation without modification of microglial functions and no apparent adverse effect in healthy adult mice (Elmore et al., 2015). CSF1R inhibition reduces neuroinflammation leading to improved disease phenotype in several mouse models of neurodegenerative diseases including Alzheimer’s disease and multiple sclerosis (Dagher et al., 2015; Wieghofer et al., 2015; Beckmann et al., 2018; Sosna et al., 2018; Spiller et al., 2018). Conversely, CSF1R inhibition worsened post-ischemic outcomes in brain ischemia by increasing the production of inflammatory factors and triggering neuronal death (Jin et al., 2017).

GW2580 is an orally available selective inhibitor of the tyrosine kinase activity of CSF1R and, to a lesser extent, other related kinases such as FMS tyrosine kinase 3 (FLT3, CD135) and oncogene KIT (c-Kit, CD117). This molecule selectively inhibits microglia/monocytes proliferation (Conway et al., 2005). Earlier, Crespo et al. (2011) showed that GW2580 ameliorates disease progression in a chemically induced mouse model of multiple sclerosis associated with a decrease in macrophages and T cells infiltration, as well as a diminution in tumor necrosis factor α expression. In a mouse model of prion disease, GW2580 administration reduces neuronal damage, slows down disease progression (Gómez-Nicola et al., 2013), and increases neurogenesis (De Lucia et al., 2016). In a mouse model of Alzheimer’s disease, chronic GW2580 treatment shifts microglial response towards anti-inflammatory phenotype and improves cognitive function (Olmos-Alonso et al., 2016). In a mouse model of amyotrophic lateral sclerosis (ALS), GW2580 treatment reduces microglial proliferation in the spinal cord leading to an increase in the lifespan and better motoneurons preservation (Martínez-Muriana et al., 2016).

Using cell-specific RNA-sequencing, we have recently shown that transcriptomic response of microglia following SCI is time-dependent and is characterized by an early proliferation followed by neuroinflammatory processes (Noristani et al., 2017b). However, specific roles of proliferating microglia in SCI pathophysiology and its association with neuroinflammation, tissue preservation and functional recovery are currently unclear.

In this study, we are targeting microglia proliferation by GW2580 administration in food diet from 4 weeks before incomplete SCI to 6 weeks post-lesion. We demosntrate that GW2580 treatment inhibits microglia proliferation after SCI and is associated with reduced gliosis, reduced microcavity formation and improved recovery of fine motor function. Inhibiting microglia proliferation may thus provide a viable therapeutic approach to reduce neuroinflammation, promote tissue preservation, and motor recovery after SCI.

## Materials and Methods

### Ethics Approval

All experiments followed the French and EU regulations (EU/Directive/2010/63 of the European Parliament and Council). This study was approved by the Veterinary Services of the Department of Hérault, the regional ethic committee for animal experimentation, and the French Ministry of National Education, Higher Education and Research (CEEA-Languedoc Roussillon, authorization number 34118). All efforts were made to minimize animal number and their suffering.

### Experimental Procedures

#### Animals, Spinal Cord Injury, Post-operative Cares and Treatments

Transgenic mice expressing enhanced green fluorescent protein (eGFP) in CNS resident microglia and circulating peripheral monocytes (CX3CR1^+/GFP^) were obtained from Dr. Dan Littman (Howard Hughes Medical Institute, Skirball Institute, NYU Medical Center, New York, NY, USA) and maintained on a C57BL/6 background (The Jackson Laboratory, Bar Harbor, ME, USA; Jung et al., 2000). CX3CR1, a Gαi-coupled seven-transmembrane chemokine receptor is prominently expressed in microglia/monocytes. CX3CR1^+/eGFP^ transgenic mice express eGFP downstream of the *Cx3cr1* promoter. Mice were housed in controlled environment (hygrometry, temperature and 12 h light/dark cycle). Only female heterozygote CX3CR1^+/GFP^ mice were used in this study.

Adult mice (3 months old) were anesthetized after inhalation of 1.0%–1.5% isoflurane gas; following vertebral thoracic 9 level (T9) laminectomy a lateral spinal cord hemisection (HS) was performed under microscope using a micro knife (10315–12, fine science tools (FST)), as described previously (Noristani et al., 2016, 2017b). Lesions were done at T9 level to obtain monoplegia. Both muscles and skin over-laying the lesion area were sutured, and animals remained under visual monitoring during 2 h over the recovery period before returning them to their home cages. Bladders were emptied manually twice daily until recovery of full sphincter control. Bodyweights were monitored prior to surgery and then daily throughout the study.

##### GW2580

Treatment started 1 month prior to injury (i.e., 2 months of age) and ended 6 weeks post-lesion corresponding to the end of the experiment. Mice were fed with a standard rodent chow (A04, maintenance diet, SAFE diet, AUJY, France) or the same regular diet containing 0.1% GW2580 (LC Laboratories, Woburn, MA, USA). To incorporate GW2580 within the chow, regular diet was mixed with water to make a dough, GW2580 was then incorporated within the dough, pellets were reconstituted and dehydrated overnight at 37°C overnight. Regular diet went through the same processes (mixed with water and reconstituted pellets) but without GW2580 incorporation.

#### *In vivo* Analysis

##### Behavioral Assessments

Mice underwent behavioral (open field and CatWalk™) assessments at 2 weeks, 1 week and 1 day prior to injury followed by 3 days, 5 days and then once a week up to 6 weeks after lesion (*n* = 12 for untreated and GW2580 groups).

##### Open Field

Spontaneous motor activity was monitored using the open field test. Mice were placed in an empty test arena (50 × 50 cm) and their movements were recorded by a camera. We measured total distance, percentage of the time spent and time of immobility (defined as no movement for more than 2 s) in two areas of the open field (center region size was designated as 20 × 20 cm and periphery corresponds to the remaining area). All behavioral analyses were done using Ethotrack (Innovationnet, Tiranges, France).

##### CatWalk™

Dynamic walking pattern analysis was done using CatWalk™ (Noldus, Wageningen, Netherlands). We analyzed data with CatMerge (InnovationNet, Tiranges, France), as previously described (Gerber et al., 2012, 2013; Noristani et al., 2018a,b). Six runs per animal were recorded at each time-point with a minimum inclusion criterion of three runs crossed at the same speed and at least 3-full step sequence patterns per mice per time point. To avoid bias due to stress, we placed all mice on the CatWalk™ glass plate for 10 min 7 days prior to the first recording session. Several parameters were quantified including the “swing phase” that corresponds to the duration of no contact of the paw with the glass plate over one full step cycle, the “maximum contact” that is the time at which the largest part of a paw contacts the glass plate, the “maximum contact at %” that represent the time of maximum contact relative to stand for individual paw placements and the “paw angle” that corresponds to the angle made by front limbs (mean of both front paws) relative to the walking direction.

##### *In vivo* T2-Weighted Magnetic Resonance Imaging (T2W-MRI)

T2W-MRI acquisitions were done at 4 and 6 weeks after injury using a 9.4 Tesla apparatus (Agilent Varian 9.4/160/ASR, Santa Clara, CA, USA) equipped with a MAGNEX TS1276D, a Quadrature Volume Coils 400 MHz RF43 (43 mm internal diameter, Rapid Biomedical, Rimpar, Germany) and associated with a VnmrJ Imaging acquisition system (Agilent, Palo Alto, CA, USA), as previously described (Noristani et al., 2015, 2018a).

Briefly, mice were anesthetized (1.5% isoflurane) using a MR-compatible anesthetic equipment (*Minerve* Siemens A.G., Erlangen, Germany) and animal holder system RS2D (Haguenau, France). Respiration and body temperature were continuously assessed using a MR-compatible small animal monitoring and gating system (Model 1025, SA Instruments, Inc., Stony Brook, NY, USA). Respiration was maintained around 30–35 breaths/minute by adjusting isoflurane level and oxygen flow rate.

Axial images were obtained with spin-echo T2-sequences (MEMS, Multi Echo Multi Slices) protocol using the following parameters: repetition time (TR) = 1,200 ms; echo time (TE) = 10 ms; (number of echoes) NE = 2; (average) AVG = 4; (field of view) FOV = 30 × 30 mm; 28 slices; thickness = 0.6 mm; gap = 0 mm; acquisition *matrix* (N_READ_ × N_PHASE_) = 256 × 256. All image acquisitions were synchronized with respiration to reduce motion artifacts, repetition time depended on breath period (T_BREATHE_), typically about 2 s. Consequently, the scanning time for axial image acquisition was approximately 35 min (T_BREATHE_ × N_PHASE_ × AVG). All MRI visualizations and segmentations were done manually using Myrian Software (Intrasense, Montpellier, France) by delineating intact (entire spinal cord, white and gray matters) and damaged tissues, respectively.

#### *Ex vivo* Analysis

At 6 weeks post-injury, immediately after the last *in vivo* MRI acquisition, mice were not awakened and were injected with a lethal dose of tribromoethanol (500 mg/kg, Sigma-Aldrich). Mice were then transcardially perfused using cold 0.1 M phosphate buffer saline (PBS) at pH 7.2 followed by cold 4% paraformaldehyde (Sigma Aldrich, Gilligham, UK) in 0.1 M PBS. The entire spinal cords were then dissected and post-fixed in the same fixative for two additional hours. Tissues were then stored in 1% paraformaldehyde until *ex vivo* MRI acquisition.

##### *Ex vivo* T2W-MRI

Dissected spinal cords were positioned in a 4-mm-diameter glass tube filled with fluorinert FC-770 liquid (3 M™ Electronic Liquids, Saint Paul, USA) surrounded by a custom-made solenoid coil devoted to SCI experiments (Coillot et al., 2016). Fluorinert (a proton-free fluid with low water solubility and similar susceptibility to the tissue) minimizes background noise from the surrounding medium during image acquisition. The tube was then placed in the 9.4 Tesla apparatus for *ex vivo* MRI acquisition. The following acquisition parameters were used for T2W-MRI: spin-echo multi-slices (SEMS); TR = 1,580 ms, TE = 30.55 ms, NE = 1, AVG = 30, FOV = 10 × 10, 36 slices, thickness = 1 mm, gap = 0 mm, acquisition matrix = 128 × 128, scanning time: approximately 100 min. Data were processed using a MATLAB-based in-house toolbox. All MRI segmentations were done manually using Myrian software (Intrasense, Montpellier, France).

Following *ex vivo* MRI, spinal cords were placed in 30% sucrose in 0.1 M PBS for cryoprotection, included in Tissue-Tek (Sakura, Alphenaanden Rijn, Netherlands), frozen and kept at −20°C until cutting.

##### Histology

Spinal cords were transversally cut at 14 μm thickness and sections were collected on Superfrost Plus slides using a cryostat (Microm HM550, Thermofisher Scientific, Waltham, MA, USA). Toluidine Blue and Luxol Fast Blue/Neutral Red were used in two series of sections respectively to evaluate the overall structural integrity of the spinal cord and estimate lesion extension. For Toluidine Blue staining, axial spinal cord sections were washed twice in 0.1 M PBS, incubated in 0.1% Toluidine Blue (Sigma Aldrich, Gilligham, UK) solution for 10 min under mild shaking, followed by brief incubation in 100% ethanol for 10 s, two washes of xylene. For Luxol Fast Blue/Neutral Red staining, slides were placed in 95% ethanol (5 min) followed by incubation in 0.1% Luxol Fast Blue under mild shaking (12 h, room temperature). Slides were then rinsed in milli Q water (1 min), placed in 0.05% lithium carbonate (1 min) and washed in tap water (1 min). Subsequently, slides were incubated in 0.5% neutral red solution (10 min), 100% ethanol (5 min) and two washes of xylene (10 min each). All slides were then cover slipped using Eukitt (Sigma Aldrich, Gilligham, UK). The lesion site was identified at low magnification due to numerous small Toluidine Blue-positive nuclei surrounding the disorganized tissue. To evaluate lesion extension and volume, up to 10 sections rostral and caudal to the lesion site were analyzed at 630 μm intervals throughout the injured segment of the spinal cord centered on the injury site. The lesion area (μm^2^) was expressed as a percentage of the total surface area for each spinal cord section. Graphs representing lesion surface area along the rostro-caudal axis were used to estimate lesion volume represented by the area under the curve (AUC). Images were analyzed using NDP view software (NanoZoomer Digital Pathology System, Hamamatsu, Hamamatsu city, Japan).

Microcavities were measured in the gray matter of the spinal cord on Toluidine Blue stained sections using Photoshop software (Adobe Systems, San José, CA, USA). The surface area of microcavities were expressed as a percentage of total gray matter area. Up to five sections rostral and caudal to the lesion site were analyzed at 630 μm intervals throughout the injured segment of the spinal cord centered on the lesion site.

##### Immunohistochemistry

Axial spinal cord sections were washed twice in 0.1 M PBS then placed for 10 min in hydrogen peroxide (H_2_O_2_, 1% in 0.1 M PBS, Sigma Aldrich, Gilligham, UK). Unspecific binding sites were blocked for 2 h with 0.1 M PBS containing bovine serum albumin (BSA, 1%, Sigma Aldrich, Gilligham, UK) and Triton X-100 (0.1%, Sigma Aldrich, Gilligham, UK) and then incubated for 48 h at 4°C with primary antibody. Sections were rinsed with 0.1 M PBS 3 × 10 min and incubated in 1:500 dilution of the corresponding peroxidase-conjugated secondary antibody (Jackson Immunoresearch, Stratech Scientific Ltd., Soham, UK) for 2 h at room temperature. Sections were rinsed with 0.1 M Trizma base saline (TRIS) 3 × 10 min and the peroxidase reaction product was visualized using Pierce DAB (3,3′-Diaminobenzidine) substrate kit (Thermofisher Scientific, Waltham, MA, USA). The reaction was stopped by rinsing sections in 0.1 M TRIS for 3 × 10 min. Sections were then dehydrated in ascending ethanol concentration (70, 80, 95% and absolute) followed by xylene for 10 min each. Cover slips were applied using Eukitt (Sigma Aldrich, Gilligham, UK) and slides were left to dry overnight. For each staining, negative controls (same protocol but omitting primary antibody) were done in parallel, which resulted in no staining (results not shown).

Antibodies used were rabbit anti-IBA1 (Wako Pure Chemical Industries, Osaka, Japan; 1:1,000) and rabbit anti-GFAP (Dako, Glostrup, Denmark; 1:1,000). To avoid bias due to experimentation, immunostainings of all animals were done simultaneously for a given time point and protein.

Optical density (OD) was measured at different distances from the lesion site using ImageJ software (National Institutes of Health, Bethesda, MD, USA), as described previously (Mukaino et al., 2010). Non-specific background was determined for each section by subtracting the OD obtained from the background without tissue. OD quantifications included gray and white matters (excluding the dorsal funiculus) as well as the dorsal funiculus of the spinal cord. Dorsal funiculus is a prominent zone for microglia/monocytes activation after SCI. Indeed, we and others have shown that high density of activated microglia/monocytes within the dorsal funiculus after SCI (Wu et al., 2005; Noristani et al., 2017a; Le Corre et al., 2018). Damaged tissue at the lesion epicenter was excluded. OD results for GW2580-treated animals were expressed as percentage of OD values of untreated mice for each protein at a given time point.

#### Bromodesoxyuridin (BrdU) Analysis

To evaluate GW2580 effect on microglia/monocytes proliferation in both uninjured and injured mice, we added additional groups of animals that were administered with BrdU (i.p., 100 mg/kg, Sigma Aldrich, Gilligham, UK, in sterile saline). Mice were divided into four different groups: uninjured reconstituted normal diet (*n* = 3); uninjured GW2580 diet (*n* = 4), SCI reconstituted normal diet (*n* = 5), and SCI GW2580 diet (*n* = 5). As previously, GW2580 treatment started 1 month prior to surgery. The first BrdU injection started immediately after SCI and continued daily over a 1-week period. Mice were sacrificed at 2 weeks after SCI and their spinal cords underwent *ex vivo* MRI as described above followed by Toluidine Blue histological and immunohistochemical analyses. For BrdU detection, sections were pre-incubated in 2 N hydrogen chloride (HCl) for 30 min for deoxyribonucleic acid (DNA) denaturation followed by 0.1 M sodium borate at pH 8.5 buffer washes (Sigma Aldrich, Gilligham, UK) for 3 × 10 min. Slides were then incubated with a combination of chicken anti-GFP (1:1,000) and rat anti-BrdU antibody (1:500; both from Abcam, Cambridge, UK) and then rinsed with PBS 3 × 10 min. Slides were then incubated in Alexa 488 and 594 secondary antibodies (both 1:1,000, Life Technologies, Carlsbad, CA, USA). Sections were cover slipped using fluorescence mounting medium (Dako, Glostrup, Denmark).

BrdU/eGFP double positive cells were identified and manually counted using ImageJ software (National Institutes of Health, Bethesda, MD, USA). Briefly, section containing the epicenter and sections located 3.2 mm rostral and 1.3 mm caudal to the epicenter were used for analysis. Counting was done using the Multi-Point tool in ImageJ.

##### Microscopy and Quantifications

All images were obtained using NanoZoomer RS slide scanner that uses constant light intensity and exposure time (NanoZoomer Digital Pathology System and NDP view software, Hamamatsu City, Japan). NDP view software was used to export images at a constant magnification. All images were exported with the same parameters for all sections in each immunohistochemistry and time points. All quantifications were done blindly. High magnifications were obtained using a bright-field up-right microscope (Leica DM1000 LED, Mannheim, Germany). For immunofluorescence images, we used laser scanning confocal microscopy (Leica SPE, Mannheim, Germany). Laser intensity and detector sensitivity settings were kept constant for all image acquisitions within a given experiment.

#### Statistics

Two-way analysis of variance (ANOVA) with Bonferroni *post hoc* test was used for multiple comparisons between untreated and GW2580-treated mice in BrdU experiment, MRI experiments and behavioral analysis. One-way ANOVA with Tukey *post hoc* test was used to compare functional recovery between untreated and GW2580-treated mice in behavioral assessment (open field and CatWalk™). Unpaired *t*-tests were also used to compare lesion extension and AUC in MRI and Toluidine Blue experiments as well as microcavities and OD quantifications.

Finally, non-parametric Spearman’s rank-order correlation was used for correlation analyses between *ex vivo* T2W-MRI and Toluidine Blue data as well as between Toluidine Blue and Luxol Fast Blue/Neutral Red data (confidence interval 95%, two-tailed). Significance was accepted at p ≤ 0.05. Data were analyzed using GraphPad Prism 5.0 (GraphPad Software Inc., La Jolla, CA, USA). All data are displayed as the mean ± standard error of the mean (SEM).

## Results

### CSF1R Blockade Limits Microglia Proliferation Following Spinal Cord Injury

**Figure 1:**
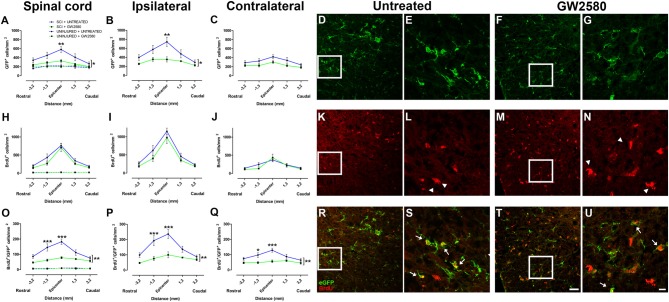
Quantitative analysis of microglia and proliferative cells 2 weeks after spinal cord injury (SCI). Densities of enhanced green fluorescent protein (eGFP)-positive cells (microglia; **A–G**), BrdU-positive cells (proliferative cells; **H–N**) and BrdU/eGFP-positive cells (proliferative microglia; **O–U**) from spinal cords of untreated and GW2580-treated mice. Quantifications were done at the epicenter as well as at 3.2 and 1.3 mm distances rostro-caudal to the lesion epicenter in the whole spinal cord **(A,H,O)**, the ipsilateral side **(B,I,P)** and the contralateral side **(C,J,Q)** in untreated and GW2580-treated mice. Values for uninjured untreated and uninjured GW2580-treated mice are represented as dashed line **(A,H,O)**. Data are expressed as number of cells per square millimeters. Data are mean ± standard error of the mean (sem) per section per group. Two-way analysis of variance (ANOVA; *p < 0.05; **p < 0.01) with a *post hoc* Bonferroni multiple comparison test to compare injured groups (GW2580-treated and untreated) **p < 0.01; ***p < 0.001. *n* = 5 for each group. Confocal micrographs displaying eGFP **(D–G)**, BrdU **(K–N)**, and dual BrdU/eGFP **(R–U)** expression. Confocal micrographs confirming BrdU incorporation into non-microglial cells (arrowheads in **L,N**) and BrdU incorporation into proliferating microglia (arrows in **S,U**). Enlargement of the boxes in **(D,F,E,G)**, respectively. Enlargement of the boxes in **(K,M,L,N)** respectively. Scale bar = 20 μm **(D,F,K,M,R,T)** and 10 μm **(E,G,L,N,S,U)**.

To study the effect of microglia depletion in SCI context, we orally administered GW2580 to CX3CR1^+/eGFP^ mice subjected to a lateral HS of the spinal cord. Initially, using BrdU injections, we investigated the effect of CSF1R blockade on microglia number and proliferation in both non-injured animals and in injured mice at 2 weeks after lesion. In non-injured mice, immunohistochemistry using anti-BrdU showed no effect of GW2580 treatment, indeed, no difference in the density of total microglia (eGFP^+^ cells), proliferating cells (BrdU^+^ cells) and proliferating microglia (eGFP^+^/BrdU^+^ cells) was observed between treated and untreated animals (Figures 1A,H,O and Supplementary Figures S1A–I, dashed lines). Two weeks following SCI, microglia, proliferating cells and proliferating microglia were counted in untreated (Supplementary Figures S2A–I and Figures 1A–E,H–L,O–S) and GW2580-treated mice (Figures 1A–C,F–J,M–Q,T,U and Supplementary Figures S2J–R). Quantifications were done rostral (Supplementary Figures S2A–C,J–L) and caudal (Supplementary Figures S2G–I,P–R) to the lesion site as well as within the lesion site and contralateral to the lesion at the epicenter (Supplementary Figures S2D–F,M–O). We observed a significant decrease in eGFP^+^ cells density in the GW2580-treated group as compared to the untreated group in the entire spinal cord (Figure 1A) and on the ipsilateral side (Figure 1B) but not on the contralateral side (Figure 1C). No significant difference between untreated and treated groups was observed in BrdU^+^ cells density in the entire spinal cord (Figure 1H), including both ipsilateral (Figure 1I) and contralateral (Figure 1J) sides of the spinal cord. However, the density of eGFP^+^/BrdU^+^ cells was significantly reduced in the GW2580-treated as compared to untreated group (Figures 1O–Q). To provide a more in-depth analysis, we compared cell distribution in the gray (Supplementary Figures S1A,D,G) and the white matters (Supplementary Figures S1B,E,H) as well as in the dorsal funiculus (Supplementary Figures S1C,F,I). The density of eGFP^+^ cells was significantly decreased by GW2580 treatment in the white matter (Supplementary Figure S1B) and in the dorsal funiculus (Supplementary Figures S1C) but not in the gray matter (Supplementary Figure S1A). BrdU^+^ cells were un-affected by the treatment (Supplementary Figures S1D–F). Contrariwise, the density of eGFP^+^/BrdU^+^ cells was decreased in GW2580-treated mice throughout the spinal cord (Supplementary Figures S1G–I). This decrease in eGFP^+^/BrdU^+^ cells is maximal at the epicenter and its rostral vicinity reaching up to 60% reduction (from 145 and 183 cells/mm^2^ in untreated to 62 and 78 cells/mm^2^ in the treated group, rostral and at the epicenter, respectively Figure 1O).

Taken together, these findings demonstrate that GW2580 treatment: (1) did not modify the density of microglia, proliferating cells and proliferating microglia in non-injured animals; (2) decreased proliferating microglia throughout the spinal cord in an injury context; and (3) decreased total microglia within the white matter and the dorsal funiculus in an injury context. Thus, CSF1R blockade using GW2580 specifically inhibits microglial proliferation after SCI.

### Reduced Microglia Proliferation Ameliorates Fine Motor Recovery After Lateral Hemisection of the Spinal Cord

**Figure 2:**
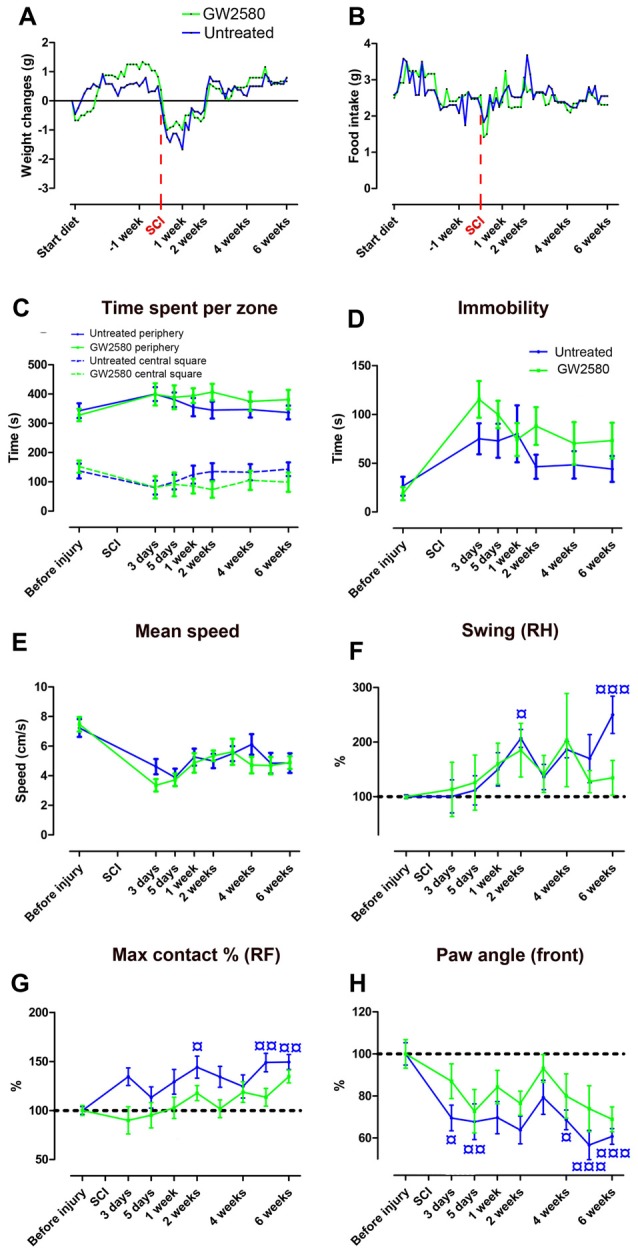
Effects of GW2580 treatment on spontaneous motor activity and gait patterns after SCI. Line curves displaying weight changes **(A)**, food intake **(B)** and spontaneous motor activity **(C–E)** in untreated and GW2580-treated groups. Open field analysis **(C–E)** demonstrated no difference between untreated and GW2580-treated groups in time spent per zone (central square and periphery, **C**), total time of immobility **(D)** and mean speed **(E)** over 6 weeks after SCI. Line graphs showing better dynamic walking pattern recovery in GW2580-treated as compared to untreated group using CatWalk™ apparatus **(F–H)**. Parameters analyzed include swing of the contralateral right hind (RH) paw (Swing RH, **F**); “Max contact at %” of the right front (RF) paw (Max contact % RF, **G**) and paw angle of the front paws (mean of both front paws; **H**). Data are expressed as mean ± SEM per time point per condition. Two-way ANOVA: ns (non-significant) in all graphs. One-way ANOVA followed by Tukey’s multiple comparison *post hoc* test to compare the evolution overtime within group ^¤^*p* < 0.05; ^¤¤^*p* < 0.01; ^¤¤¤^*p* < 0.001. *n* = 12 for each group.

We next investigated the putative effect of GW2580-mediated CSF1R inhibition and reduced microglial proliferation on motor behavior following SCI. GW2580 treatment had no effect on animal weight (Figure 2A) and food intake (Figure 2B) both prior and after injury. Open field analysis over 6 weeks post-injury follow-up revealed no difference in anxiety behavior between untreated and treated groups as reflected by the equivalent time spent per zone (central and periphery, Figure 2C) and their time of immobility (Figure 2D). Spontaneous motor activity after SCI analyzed by the mean speed (Figure 2E) was also unaffected by GW2580 treatment. Dynamic walking patterns analysis using CatWalk™ confirmed that the mean speed to cross the glass plate was similar between untreated and GW2580-treated mice (data not shown). CatWalk™ analysis also allows detailed examination of multiple parameters for individual footprints. There is no significant difference in the ipsilateral hind paw (left hind), suggesting abnormal paw placement even in GW2580-treated animals that may prevent to reach significance (data not shown). Subsequently, we examined other paws (right hind (RH), right front (RF) and both front paws) to evaluate fine motor recovery in both groups. Direct comparison of the swing phase of the contralateral RH paw of the two groups revealed no difference (two-way ANOVA). However, evolution over time was significantly modified by the injury in the untreated group (one-way ANOVA, *p* < 0.001, Figure 2F) conversely to the GW2580-treated group (one-way ANOVA, ns, Figure 2F) where the swing value remained similar to before surgery (100%). Similarly, “max contact at %” of the RF paw significantly increased following SCI as compared to before injury in the untreated but not in GW2580-treated group (one-way ANOVA, *p* < 0.001, Figure 2G). Likewise, the front limbs paw angle remained un-affected by SCI in the GW2580-treated but not in the untreated group (one-way ANOVA, *p* < 0.001, Figure 2H). Thus, in the untreated group all parameters associated with fine motor recovery (Figures 2F,G) were affected, conversely to the GW2580-treated group.

These findings first demonstrate that GW2580 treatment in both un-injured and injured mice have no effect on anxiety behavior and gross motor recovery. Second, following SCI, GW2580 treatment improved placement of contralateral hind paw and front paws over the recovery phase. Treated animal do not compensate to achieve similar speed as the untreated animal. Altogether, these results suggest that GW2580 treatment induces a better recovery of fine motor function after SCI.

### CSF1R Inhibition Has no Effect on Lesion Extension and Volume After SCI

**Figure 3:**
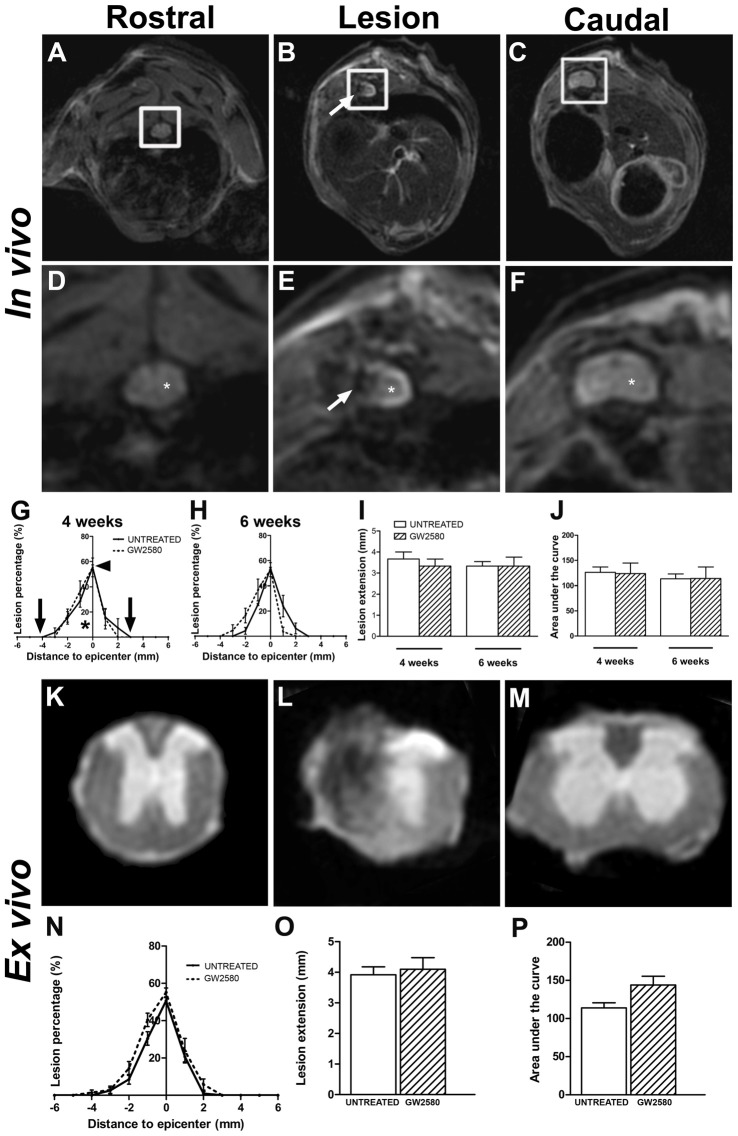
*In vivo* and *ex vivo* magnetic resonance imaging (MRI) assessments of the lesion evolution in untreated and GW2580-treated mice after SCI. *In vivo* axial sections taken rostral **(A,D)**, within **(B,E)** and caudal **(C,F)** to the lesion epicenter at 6 weeks after SCI in GW2580-treated mouse. Spinal cord axial images zoomed in on the spinal cord **(D–F)** corresponding to *in vivo* MRI (white insets in **A–C**). Arrows in **(B,E)** indicate hypo-intense signal on the hemisected side of the spinal cord. Asterisk in **(D,F)** indicate hyper signal of the gray matter. Quantification of the lesion surface area, extension and volume in untreated and GW2580-treated groups at 4 **(G,I,J)** and 6 weeks **(H,I,J)** after SCI. Arrowhead in **(G)** indicates the percentage of damage tissue at the lesion epicenter. Arrows in **(G)** indicates lesion extension along the rostro-caudal axis. *Ex vivo* axial MRI taken rostral **(K)**, within **(L)** and caudal **(M)** to the lesion site at 6 weeks after SCI in GW2580-treated mice. Quantification of the lesion surface area **(N)**, extension **(O)** and volume **(P)** in untreated and GW2580-treated groups at 6 weeks after SCI. Data are expressed as mean ± SEM per group and per time-point. Two-way ANOVA **(I,J)**: ns, Student’s unpaired *t*-test **(O,P)**: ns. *In vivo* MRI *n* = 6 for each group; *ex vivo* MRI *n* = 12 for each group.

To examine the potential effect of reduced microglia proliferation on lesion evolution following injury, we carried out longitudinal *in vivo* T2W-MRI analysis in GW2580-treated and untreated groups at 4 and 6 weeks after injury (Figures 3A–F). Altered tissues were identified by a hypo-intense signal at the perihematomal region on the hemisected side of the spinal cord (Figures 3B,E, arrows) that contrasts with the normal hyper-intense signal of the contralateral gray matter observed on the opposite side of the lesion as well as rostral and caudal to the lesion (Figures 3D–F, asterisk). The lesion epicenter was identified as the section with the highest percentage of damaged tissues. Comparisons of GW2580-treated and untreated groups showed no difference in the percentage of damage tissues at the lesion epicenter (Figure 3G, arrowhead) and lesion extension on the rostro caudal axis (Figure 3G, in between the two arrows and Figure 3I) at 4 (Figure 3G) and 6 weeks (Figure 3H) after spinal cord HS. No difference in lesion evolution was seen between groups (two-way ANOVA) as well as overtime in each of the group (one-way ANOVA). Lesion volume was estimated by calculating the AUC (Figure 3G; asterisk); both groups displayed similar lesion volumes at both 4 and 6 weeks following injury (Figure 3J). To discriminate more precisely between the intact and the injured spinal cord tissues, we acquired *ex vivo* T2W-MRI of the spinal cords at 6 weeks post injury corresponding to the end of the *in vivo* experiment (Figures 3K–M) and at 2 weeks post-injury corresponding to the group of mice subjected to BrdU experiments (Supplementary Figures S3A–C). No difference in any of the parameters was observed between untreated and GW2580-treated groups both at 2 (Supplementary Figures S3A–C) and 6 weeks after injury (Figures 3N–P).

**Figure 4:**
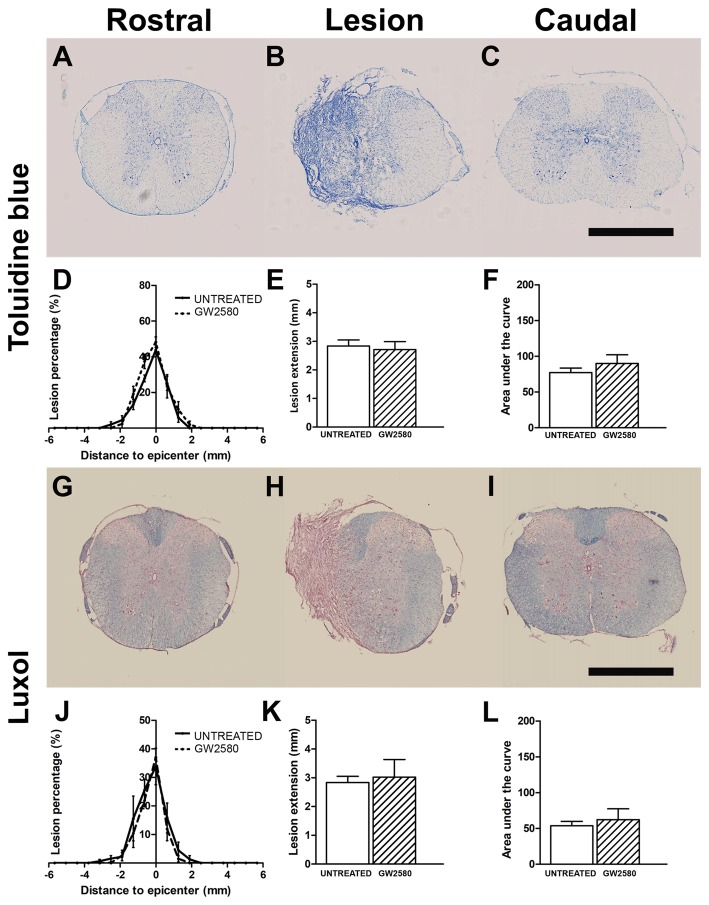
Lesion assessment at 6 weeks after SCI using histological staining. Bright-field micrographs displaying Toluidine Blue stained axial sections rostral **(A)** within **(B)** and caudal **(C)** to the lesion site at 6 weeks after SCI in GW2580-treated mice. Toluidine Blue-based quantification of the lesion surface area **(D)**, extension **(E)** and volume **(F)** in untreated and GW2580-treated groups at 6 weeks after SCI. Luxol Fast Blue/Neutral Red stained axial sections rostral **(G)**, within **(H)** and caudal **(I)** to the lesion site at 6 weeks after SCI in GW2580-treated mice. Luxol Fast Blue-based quantification of the lesion surface area **(J)**, extension **(K)** and volume **(L)** in untreated and GW2580-treated groups at 6 weeks after SCI. Student’s unpaired *t*-test: ns. Data are expressed as mean ± SEM per condition. *n* = 12 for each group. Scale bar = 1 mm.

We then quantified lesion extension and volume using more classical methods than MRI; we indeed performed Toluidine Blue staining in the same spinal cords that underwent T2W-MRI at 2 weeks (Supplementary Figures S3D–F) and 6 weeks after injury (Figures 4A–C). We also performed Luxol Fast Blue/Neutral Red staining on the same samples at 6 weeks post-lesion (Figures 4G–I). In line with *in vivo* and *ex vivo* MRI findings, using Toluidine Blue and Luxol Fast Blue/Neutral Red stainings, we confirmed no difference between untreated and GW2580-treated mice in percentage of damage tissues at the epicenter, lesion extension and lesion volume at 2 (Supplementary Figures S3D–F) and 6 weeks post-injury (Figures 4D–F,J–L). Comparison of *ex vivo* MRI and Toluidine Blue analyses showed good correlation between the two modalities (untreated group at 2 weeks: *r* = 0.84; GW2580-treated group at 2 weeks: *r* = 0.92; untreated group at 6 weeks: *r* = 0.90; GW2580-treated group at 6 weeks: *r* = 0.88; Supplementary Figures S3G,H). At 6 weeks comparison of Toluidine Blue and Luxol Fast Blue/Neutral Red analyses also presented good correlation between modalities (untreated group at 6 weeks: *r* = 0.99; GW2580-treated group at 6 weeks: *r* = 0.91, data not shown).

Taken together, these findings demonstrate that GW2580 treatment did not modify lesion extension and volume over a period of 6 weeks following spinal cord HS.

### Reduced Microglia Proliferation After SCI Limits Overall Glial Reactivity

**Figure 5:**
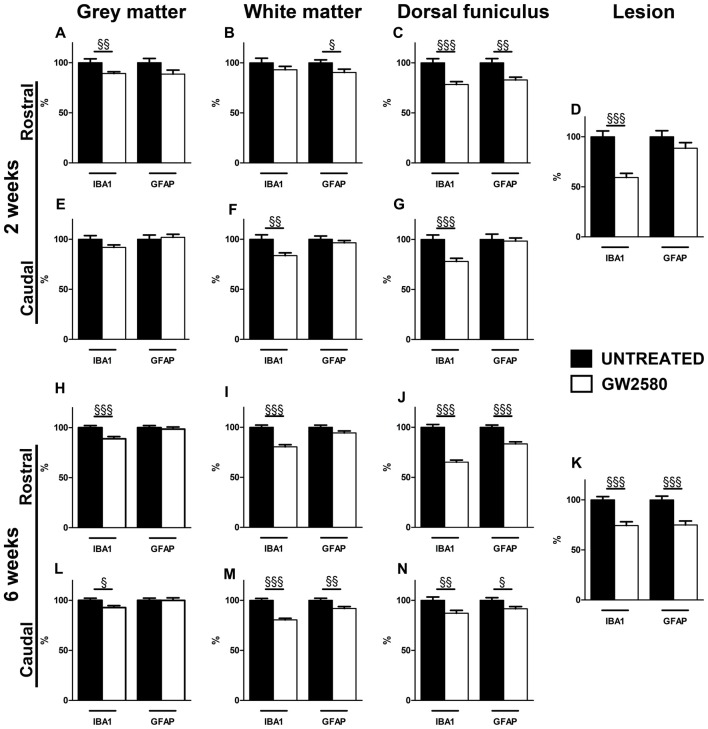
Effect of GW2580 on gliosis after SCI. Bar graphs displaying IBA1 and GFAP immunostaining optical densities in GW2580-treated (white bar graphs) and untreated mice (black bar graphs) at 2 **(A–G)** and 6 weeks after SCI **(H–N)** in the gray matter **(A,E,H,L)**, the white matter (excluding the dorsal funiculus, **(B,F,I,M)**), in the dorsal funiculus **(C,G,J,N)** and within the lesion site **(D,K)**. The Optical density (OD) values of GW2580-treated mice are expressed as a percentage of OD obtained from untreated mice (black bars). Quantifications were done in the spinal cord at 5.7 mm distance rostro-caudal to the lesion epicenter. Data are expressed as mean ± SEM per condition. Student’s unpaired *t*-test ^§^*p* < 0.05, ^§§^*p* < 0.01, ^§§§^*p* < 0.001. *n* = 12 for each group, marker and time point.

**Figure 6:**
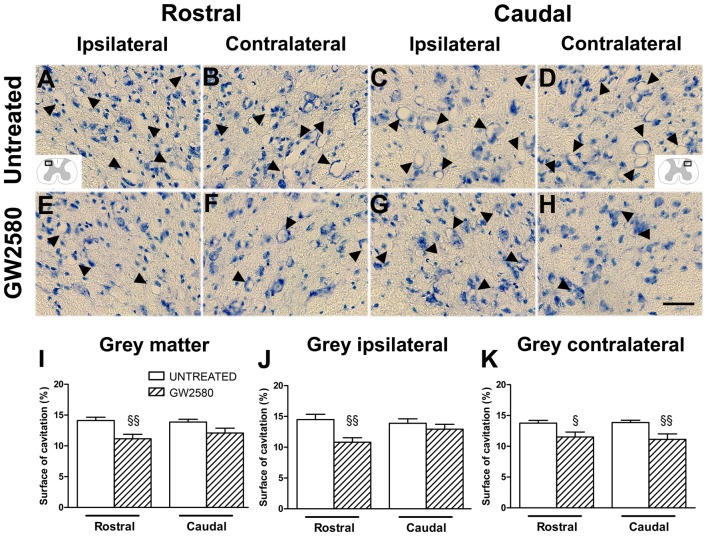
GW2580-induced decrease in microcavity formation at 6 weeks after SCI. Micrographs of Toluidine Blue stained axial spinal cord sections at high magnification displaying microcavities (arrowheads) in the dorsal horn of untreated **(A–D)** and GW2580-treated mice **(E–H)** taken rostral (ipsilateral **(A,E)** and contralateral **(B,F)**) and caudal (ipsilateral **(C,G)** and contralateral **(D,H)**) to the lesion site. Schematic drawings in **(A,D)** display the field of view where images were taken from (ipsilateral and contralateral dorsal horn). Bar graphs display percentages of cavitation in the total gray matter of untreated and GW2580-treated mice at 6 weeks after SCI **(I–K)**. Cavitations were expressed as a percentage of the total gray matter surface area at 2.5 mm distance rostro-caudal to the lesion epicenter in the whole spinal cord **(I)**, the ipsilateral side **(J)** and the contralateral side **(K)**. Data are expressed as mean ± SEM per condition. Student’s unpaired *t*-test ^§^*p* < 0.05, ^§§^*p* < 0.01. *n* = 12 for each group. Scale bar = 40 μm.

To investigate the impact of reduced microglia proliferation at a cellular level, we first quantified glial reactivity following SCI using IBA1 (a microglia/macrophage marker, Supplementary Figures S4, S5) and GFAP (an astrocytic marker) immunostainings (Supplementary Figures S6, S7). We analyzed untreated and GW2580-treated mice at 2 (Figures 5A–G) and 6 weeks (Figures 5H–N) after lateral HS of the spinal cord. To determine SCI-induced changes in protein expression, the mean OD was measured at the lesion site, rostral and caudal to the lesion site in all groups.

Injured GW2580-treated group displayed a decrease in IBA1 immunoreactivity both rostral and caudal to the lesion in the gray matter, the white matter, the dorsal funiculus and within the lesion area as compared to untreated (Figure 5 and Supplementary Figures S4, S5). At 2 weeks after SCI, the GW2580-treated group showed a decrease in microglia reactivity rostral to the lesion epicenter in the gray matter (Figure 5A and Supplementary Figures S4A,D), caudal to the epicenter in the white matter (Figure 5F and Supplementary Figures S4C,F,I,L) and on both sides of the lesion in the dorsal funiculus (Figures 5C,G and Supplementary Figures S4A,D,C,F). This decrease in microglia reactivity was also observed within the lesion site (Figure 5D and Supplementary Figures S4B,E,H,K). By 6 weeks after injury, GW2580-induced widespread decrease in IBA1 immunoreactivity compared to untreated group throughout the spinal cord, within the lesion site as well as both rostral and caudal to the lesion site (Figures 5H–N and Supplementary Figure S5). Morphologically, the IBA-1-positive microglia/macrophages included: (1) ramified/resting cells with small cell bodies and randomly distributed thin-to medium-sized processes (Supplementary Figures S5J,L, arrows); and (2) reactive/hypertrophied microglia/macrophages displaying enlarged soma with short/thick processes (Supplementary Figures S5G,I, arrowheads). Whilst untreated group displayed both ramified and hypertrophic microglia/macrophages (Supplementary Figures S5G–I), GW2580-treated group predominantly showed ramified microglia, further confirming reduced microglia/macrophage reactivity (Supplementary Figures S5J–L).

Injured GW2580-treated group also displayed a slight decrease in GFAP immunoreactivity as compared to untreated animals (Figure 5 and Supplementary Figures S6, S7). At 2 weeks after SCI, the GW2580-treated group showed a decrease in astrocytic reactivity rostral to the lesion epicenter in the white matter (Figure 5B and Supplementary Figures S6A,D,G,J) and the dorsal funiculus (Figure 5C and Supplementary Figures S6A,D). By 6 weeks after injury, quantitative analysis revealed a significant decrease in GFAP immunoreactivity in the GW2580-treated group as compared to untreated group caudal to the lesion epicenter in the white matter (Figure 5M and Supplementary Figures S7C,F,I,L) and both rostral and caudal to the lesion in the dorsal funiculus (Figures 5J,N and Supplementary Figures S7A,D,C,F). This decrease in astrocytic reactivity is also observed within the lesion (Figure 5K and Supplementary Figures S7B,E,H,K). Morphologically, reactive astrocytes displayed increased GFAP expression with elongated morphology (Supplementary Figures S6G, arrowheads). Untreated group showed particular increase in reactive astrocytes within the white matter rostral to the lesion epicenter (Supplementary Figure S6G) compared to GW2580-treated mice (Supplementary Figure S6J), further confirming reduced astrocytic reactivity.

Altogether, these data suggest that the GW2850-mediated inhibition of microglia proliferation in a SCI context decreases microglia/macrophage and astrocytic reactivity. In all cases, observed decreases in expression were more pronounced at 6 weeks than 2 weeks after SCI.

### CSF1R Inhibition Reduces Microcavity Formation After SCI

Given that GW2580-mediated inhibition of microglial proliferation reduced gliosis, we finally examined its consequences on tissue preservation after lateral HS (Figure 6). We assessed the surface area occupied by microcavities in Toluidine Blue stained sections at 6 weeks after SCI both rostral (Figures 6A,B,E,F) and caudal (Figures 6C,D,G,H) to the lesion site in the gray matter of untreated (Figures 6A–D) and GW2580-treated (Figures 6E–H) groups. We identified a decrease in microcavities formation within the whole gray matter in GW2580-treated as compared to untreated group rostral to the lesion site (Figure 6I). To deepen our analysis, we evaluated separately both the ipsilateral and the contralateral sides of the spinal cord. Rostral to the lesion, GW2580 treatment induced decrease in microcavity formation both ipsilateral and contralateral to the lesion (Figures 6J,K). However, caudal to the lesion, GW2580 treatment reduced microcavity formation only contralateral to the lesion (Figures 6J,K).

Altogether, these results show that GW2580 treatment reduced microcavities formation, suggesting that inhibiting microglia proliferation after injury reduces gliosis and promotes tissue preservation.

## Discussion

In the present study, we targeted microglia proliferation in a SCI context via pharmacological inhibition of CSF1R using *per os* delivery of GW2580. We carried out detailed behavioral, MRI and histopathological analyses in CX3CR1^+/GFP^ mice. GW2580 selectively inhibited microglia proliferation and improved fine motor recovery in the injured animals. Behavioral improvement post-SCI was not associated with a decrease in lesion extension and volume assessed using *in* and *ex vivo* T2 weighted MRI as well as Toluidine Blue staining. However, CSF1R blockade resulted in reduced gliosis and better tissue preservation, suggesting a positive role of GW2580-treatment on SCI pathophysiology.

These findings thus demonstrate that reduction of microglia proliferation after SCI improves motor recovery and is associated with reduced gliosis and preserved histopathology of the injured spinal cord.

### GW2580 Treatment Specifically Inhibits Microglia Proliferation After SCI

Microglia, the resident macrophages of the CNS are the first reactive actors of the immune response after a lesion (Davalos et al., 2005; David et al., 2018). Activated microglia not only proliferate and migrate to the injury site, but also participate in the recruitment and infiltration of peripheral macrophages (David and Kroner, 2011). Microglia display a higher level of proliferation than peripheral macrophages early after SCI (Greenhalgh and David, 2014) that precedes their established role in orchestrating neuroinflammation at more chronic stages (Noristani et al., 2017b). Both pro- and anti-regenerative roles of microglia and monocytes have been reported following SCI (David and Kroner, 2011). In one hand, microglia promote the expression of neurotrophic factors (Lalancette-Hébert et al., 2007; Lambertsen et al., 2009) and phagocytose of cellular debris (Perrin et al., 2005) thus exerting beneficial effects following SCI (Mukaino et al., 2010). On the other hand, activated microglia promote inflammation and secondary tissue damage (David and Kroner, 2011). Furthermore, previous reports demonstrated over-expression of neurotoxic genes in microglia as early as 1 day following SCI (Kigerl et al., 2009; Hirbec et al., 2017).

Microglial response following traumatism exhibits some specificity as compared to other neurological disorders also involving microglial activation. Firstly, the breakdown of the blood spinal cord barrier caused by the lesion promotes infiltration of blood-derived macrophages, therefore a mixed population of activated microglia and peripheral inflammatory cells is present within the lesion site (Popovich et al., 1996). Secondly, conversely to neurodegenerative diseases characterized by a progressive and chronic activation/proliferation of microglia, microglial activation occurs more rapidly after a traumatic lesion (for review see, David et al., 2015; Okada, 2016). A peak in microglia activation occurred between 3 and 7 days post-spinal cord contusion in rats preceding a peak in monocyte influx and macrophage activation at 7 days (Popovich et al., 1997). Another study, also using contusion injury in rats, identified the peak of microglia/macrophages number at 48 h, predominantly within the gray matter and the dorsal funiculus (Carlson et al., 1998). Spinal cord compression in mice triggered microglia/macrophage proliferation as early as 1 day after injury that remained elevated for at least 14 days (McDonough et al., 2013). Moreover, using microglia-specific RNA-sequencing following complete and lateral HS of the mouse spinal cord, we identified an increase in the expression of proliferation-associated genes at 72 h following SCI (Noristani et al., 2017b).

Monocytes and microglia share the same lineage and have a similar pattern of protein expression. As CSF1 regulates differentiation, maintenance and proliferation of myeloid lineage cells (Patel and Player, 2009) several pharmacological agents inhibiting CSF1R had been tested in animal models of neurological disorders. PLX3397 inhibits CSF1R signaling and ablated around 99% of microglia in control adult mice (Elmore et al., 2014). Prolonged PLX3397 treatment in a mouse model of Alzheimer’s disease reduced amyloid plaque deposition and improved cognitive function (Sosna et al., 2018). However, in a transient focal cerebral ischemia and reperfusion model, PLX3397 augmented the production of inflammatory mediators by astrocytes and exacerbated brain infarction and neuro-deficits (Jin et al., 2017). Chronic treatment with other CSF1R inhibitor (PLX5622) completely ablated microglia from the retinal ganglion cells with no effect on axon regeneration after optic nerve injury (Hilla et al., 2017). Treatment using BLZ945, another brain-penetrant CSF1R inhibitor, reduced up to 60% of microglia number, whilst causing an increase in oligodendrocytes and astrocytes number as well as enhanced remyelination in several brain regions of a mouse model of multiple sclerosis without modifying the course of the disease (Beckmann et al., 2018). In a mouse model of glioblastoma multiform, BLZ945 usage increased animal survival and led to a size reduction of established tumors (Pyonteck et al., 2013).

Conversely, to a broad inhibition of microglia/macrophages, GW2580 selectively inhibits microglial proliferation (Conway et al., 2005; Gómez-Nicola et al., 2013; Olmos-Alonso et al., 2016). Oral administration of GW2580 prevented progression of Alzheimer’s disease-like pathology (Olmos-Alonso et al., 2016) and delayed the pathological course of prion disease in transgenic mouse models (Gómez-Nicola et al., 2013). In addition, GW2580 also prevented the development and treated established disease pathology in a mouse model of multiple sclerosis (Crespo et al., 2011). Furthermore, GW2580 treatment in a mouse model of ALS decreased motoneuron death, protected skeletal muscle denervation and extended the survival (Martínez-Muriana et al., 2016). Altogether, these reports, including our findings, extend the beneficial effect of GW2580 in CNS pathophysiology and trauma.

Given the rapid microglial proliferation after injury, we thus hypothesize that oral GW2580 delivery prior to SCI may be necessary to efficiently target microglia/macrophage proliferation. Using BrdU injection over 1 week starting immediately after injury, we demonstrated that GW2580 treatment specifically inhibit approximately 60% microglia proliferation at the epicenter and its rostral vicinity thus extending results already obtained in other neurological diseases including multiple sclerosis (Crespo et al., 2011), Alzheimer’s disease (Olmos-Alonso et al., 2016) and ALS (Martínez-Muriana et al., 2016). Interestingly, the decrease in microglia proliferation was not associated with a reduction in the overall number of proliferative cells (BrdU positive cells), this strongly suggest an interplay between microglia/macrophages and other cell types such as astrocytes and/or oligodendrocytes (for review see, Zhou et al., 2014). In this line, it had been reported in SCI that rapamycin, an inducer of autophagy, not only reduced macrophage/neutrophil infiltration into the injury site and microglia activation but also inhibited astrocyte proliferation (Goldshmit et al., 2015). Furthermore, a recent report using stab wound brain injury in transgenic mouse model with reduced monocyte invasion reported a strong increase in astrocyte proliferation (Frik et al., 2018). Additionally, GW2580 had been delivered orally to a mouse model of systemic lupus erythematosus that display features of lupus nephritis and neuropsychiatric systemic lupus erythematosus involving macrophages and microglia, respectively (Chalmers et al., 2017). GW2580-treated mice displayed macrophage depletion within kidneys and decreased levels of several proinflammatory cytokines associated with improved renal histopathology, attenuated disease evolution and ameliorated depression-like behavior. Surprisingly, treated-mice also presented an increase in IBA1 staining intensity in the brain cortex. One interpretation is that GW2580 modification of peripheral macrophages and microglial progenitors in the bone marrow may also indirectly modulate CNS pathologies (for review see, Han et al., 2017). However, others have shown that CSF1R inhibition does not decrease blood monocyte (Chitu et al., 2012). Furthermore, GW2580 may also play a direct role on neurons since spinal motor neurons express a low level of CSF1R that is increased 1 week after ischemic lesion of the cerebral cortex (Wang et al., 1999). Moreover, developmental deletion of CSF1R increases kainic acid-induced neurodegeneration, suggesting that neuronal CSF1R regulate neural progenitor self-renewal and survival (for review see, Chitu et al., 2016). However, in our experiments both serotonin (5HT) and growth associated protein 43 (GAP 43) staining were not different between GW2589-treated and untreated mice (data not shown).

### GW2580 Treatment Improves Fine Motor Recovery After SCI

In our study, open field analysis showed no difference in gross motor recovery, however, GW2580-treated group demonstrated improved recovery of fine motor function after incomplete SCI. Indeed, using dynamic walking pattern analyses, we observed a better fine motor recovery of the front paws and contralateral hind paw, suggesting that GW2580-treated mice do not compensate to attain similar performance over the recovery phase as the untreated animals.

Modulation of the inflammatory environment induced by SCI has been previously reported to play an important role on functional recovery. Inhibition of IL-6, a pro-inflammatory cytokine with major role in secondary damages after SCI, impeded injury-induced astrogliosis, reduced infiltration of inflammatory cells and improved motor recovery (Okada et al., 2004). Similarly, inhibition of TNFα in SCI reduced neutrophil infiltration, neuroinflammation, tissue damage and apoptosis associated with enhanced locomotor recovery (Genovese et al., 2008). Activated microglia/macrophages release high levels of IL-6 and TNFα early after injury (David and Kroner, 2011). Moreover, minocycline, an antibiotic that, amongst other functions, reduces microglial activation and macrophage recruitment in the CNS (Stirling et al., 2004), preserved motor function in rats following SCI (Lee et al., 2003). Finally, selective depletion of peripheral macrophages following spinal cord contusion in rats using intravenous injections of liposome encapsulated chlodronate significantly improved locomotion (Popovich et al., 1999).

These results, including ours, support that reducing microglia and/or related inflammatory cascade events after SCI promote functional recovery.

### GW2580 Treatment Modifies Tissue Re-organization After SCI

The only available method used to investigate *in vivo* tissues re-organization is MRI. We have previously reported that *in vivo* T2W-MRI clearly discriminate lesion extension and volume between lateral HS and complete section of the mouse spinal cord (Noristani et al., 2015). T2W-MRI *in vivo* acquisitions carried out at 4 and 6 weeks post-injury demonstrated no difference in lesion area and volume between untreated and GW2580-treated groups. These results were corroborated by high resolution *ex vivo* T2W-MRI and classical histology analyses that allows more precise discrimination between spared and injured tissues. Comparison between the two *ex vivo* modalities of analyses highlighted a strong correlation.

Our findings of reduced microglia/macrophage cell number and IBA1 expression confirms previous studies using CSF1R inhibitors in mouse models of neurological diseases (Gómez-Nicola et al., 2013; Olmos-Alonso et al., 2016). Further, histopathological analyses also revealed reduced astroglial reactivity after GW2580 treatment at 2 and 6 weeks after injury. These data are in line with previous reports highlighting the importance of microglia and astrocytes crosstalk, where microglia, the first responsive cell after injury can promote astrocytic activation (for review see, Liu et al., 2011). A recent study showed that selective genetic reduction of infiltrating monocytes after traumatic brain injury increased astrocytic proliferation, although GFAP-positive cells number in the vicinity of the scar was decreased (Frik et al., 2018). This is in agreement with our results since we observed that inhibiting microglial/monocytes proliferation after SCI led to reduced gliosis revealed by a decrease in IBA1 and GFAP expression.

Microcavity and necrotic lesion areas are composed of inflammatory cells surrounded by reactive astrocytes and are commonly observed in spinal cord injured human and rats, whilst only small cavities have been reported in mouse models of SCI (Bilgen et al., 2007; Surey et al., 2014). We demonstrated higher cavity formation in the gray matter of untreated mice as compared to treated animals. Microcavities form and expand after SCI due to vascular permeability, apoptosis, necrosis and hypoxia (Loy et al., 2002; Whetstone et al., 2003; Casella et al., 2006; Fassbender et al., 2011). Moreover, cavitations may also increase due to the clearance of the damaged tissues by microglia/macrophages. Decrease in microcavity formation may result from a maintained structure of the spinal cord given that GW2580-mediated inhibition of microglia/monocytes proliferation reduced microglial and astrocytes reactivity, as reported in other immunosuppressive therapies (Popovich et al., 1999). Others also reported reduced microcavity formation following treatment with anti-inflammatory agents including methylprednisolone and IL10 associated with improved functional recovery after incomplete SCI in rats (Bethea et al., 1999; Brewer et al., 1999; Takami et al., 2002).

In conclusion, pharmacological inhibition of microglia/macrophages proliferation through CSF1R blockade using GW2580 before lesion improved fine motor functional recovery associated with reduced gliosis and microcavity formation following incomplete SCI. Preventing microglial proliferation may thus offer a practical therapeutic approach to promote tissue preservation and motor recovery following SCI. GW2580 treatment after CNS lesion may thus represent the next rational step to evaluate its translational potential.

## Author Contributions

YG designed the project, performed majority of the experiments, analyzed the data and contributed to the writing of the manuscript. GS-M performed MRI acquisition and analysis. CB participated in behavioral acquisition and analysis as well as histological analysis. SB participated in histological analysis. CG-B participated in the design of MRI acquisition and analysis. HN designed the project and contributed to the writing of the manuscript. and FP conceptualized the research, designed the project, participated in the analysis and data interpretation, drafting the work and final approval.

## Conflict of Interest Statement

The authors declare that the research was conducted in the absence of any commercial or financial relationships that could be construed as a potential conflict of interest.

## Supplementary Material

FIGURE S1 Microglia proliferation in GW2580-treated and untreated mice: cell distribution in the spinal cord. Densities of eGFP-positive cells (microglia; **A–C**), BrdU-positive cells (proliferative cells; **D–F**) and BrdU/eGFP-positive cells (proliferative microglia; **G–I**) from the spinal cord of untreated and GW2580-treated mice. Quantifications were done at the lesion epicenter as well as at 3.2 and 1.3 mm distances rostro-caudal to the lesion epicenter in the preserved gray matter **(A,D,G)**, white matter **(B,E,H)** and in the dorsal funiculus **(C,F,I)** in untreated and GW2580-treated groups. Uninjured untreated and uninjured GW2580-treated mice are represented as dashed line in all graphs. Data are expressed as number of cells per square millimeters. Data are mean ± SEM per section per group. Two-way ANOVA (**p* < 0.05; ***p* < 0.01) with a *post hoc* Bonferroni multiple comparison test to compare injured groups (treated and untreated) ***p* < 0.01; ****p* < 0.001. *n* = 5 for each group.

FIGURE S2 GW2580 inhibits microglia proliferation in the mouse spinal cord at 2 weeks after SCI. Fluorescent micrographs of axial spinal cord sections from CX3CR1^+/eGFP^ mice. Microglia eGFP-positive cells **(A,D,G,J,M,P)**, BrdU staining **(B,E,H,K,N,Q)** and merged **(C,F,I,L,O,R)**. Axial sections from untreated **(A–I)** and GW2580-treated mice **(J–R)** at 2 weeks after SCI. Axial images from untreated mice rostral to the lesion epicenter **(A–C)**, at the lesion epicenter **(D–F)** and caudal to the lesion epicenter **(G–I)**. Axial images from GW2580-treated mice rostral to the lesion epicenter **(J–L)**, at the lesion epicenter **(M–O)** and caudal to the lesion epicenter **(P–R)**. In all images, the lesion is located on the left side of the spinal cord. Note: displayed rostral and caudal images are each located at 2 mm from the epicenter. Scale bar = 1 mm.

FIGURE S3 *Ex vivo* MRI and histological assessments of the lesion size in untreated and GW2580-treated mice at 2 weeks after SCI. *Ex vivo* axial T2-weighted MRI quantification of the lesion area, lesion extension and volume in untreated and GW2580-treated groups **(A–C)**. Toluidine Blue stained axial sections quantification of the lesion area, lesion extension and volume in untreated and GW2580-treated groups **(D–F)**. Correlation between *ex vivo* and Toluidine Blue analyses in untreated and GW2580 groups at 2 **(G)** and 6 weeks **(H)** after SCI. Data are expressed as mean ± SEM per group and per time-point. Student’s unpaired *t*-test: ns. *n* = 5 for each group.

FIGURE S4 IBA1 immunostaining in untreated and GW2580-treated mice at 2 weeks after SCI. Brightfield micrographs representing IBA1 immunostainings rostral **(A,D,G,J)**, within **(B,E,H,K)** and caudal **(C,F,I,L)** to the lesion epicenter in untreated mice **(A–C,G–I)** and in GW2580-treated mice **(D–F,J–L)** at 2 weeks after SCI. Higher magnification in untreated **(G–I)** and GW2580-treated mice **(J–L)** are corresponding to black insets in **(A–F)**. In all images, the lesion is on the left side of the spinal cord. Note: displayed rostral and caudal images are each located at 2 mm from the epicenter. Scale bar = 1 mm **(A–F)**; 100 μm **(G–L)**.

FIGURE S5 IBA1 immunostaining in untreated and GW2580-treated mice at 6 weeks after SCI. IBA1 immunostainings rostral **(A,D,G,J)**, within **(B,E,H,K)** and caudal **(C,F,I,L)** to the lesion epicenter in untreated mice **(A–C,G–I)** and in GW2580-treated mice **(D–F,J–L)** at 6 weeks after SCI. Higher magnification in untreated **(G–I)** and GW2580-treated mice **(J–L)** are corresponding to black insets in **(A–F)**. In all images, the lesion is on the left side of the spinal cord. Note: displayed rostral and caudal images are each located at 2 mm from the epicenter. Scale bar = 1 mm **(A–F)**; 100 μm **(G–L)**.

FIGURE S6 GFAP immunostaining in untreated and GW2580-treated mice at 2 weeks after SCI. Brightfield micrographs representing GFAP immunostainings rostral **(A,D,G,J)**, within **(B,E,H,K)** and caudal **(C,F,I,L)** to the lesion epicenter in untreated **(A–C,G–I)** mice and in GW2580-treated **(D–F,J–L)** mice at 2 weeks after SCI. Higher magnification in untreated **(G–I)** and GW2580-treated mice **(J–L)** are corresponding to black insets in **(A–F)**. In all images, the lesion is on the left side of the spinal cord. Note: displayed rostral and caudal images are each located at 2 mm from the epicenter. Scale bar = 1 mm **(A–F)**; 100 μm **(G–L)**.

FIGURE S7 GFAP immunostaining in untreated and GW2580-treated mice at 6 weeks after SCI. GFAP immunostainings rostral **(A,D,G,J)**, within **(B,E,H,K)** and caudal **(C,F,I,L)** to the lesion epicenter in untreated **(A–C,G–I)** mice and in GW2580-treated **(D–F,J–L)** mice at 6 weeks after SCI. Higher magnification in untreated **(G–I)** and GW2580-treated mice **(J–L)** are corresponding to black insets in **(A–F)**. In all images, the lesion is on the left side of the spinal cord. Note: displayed rostral and caudal images are each located at 2 mm from the epicenter. Scale bar = 1 mm **(A–F)**; 100 μm **(G–L)**.
