# Supplementary figures and images for: CSF1R Inhibition Reduces Microglia Proliferation, Promotes Tissue Preservation and Improves Motor Recovery After Spinal Cord Injury
Source: Front Cell Neurosci. 2018 Oct 16;12:368. doi: 10.3389/fncel.2018.00368 (PMC6198221; doi:10.3389/fncel.2018.00368)

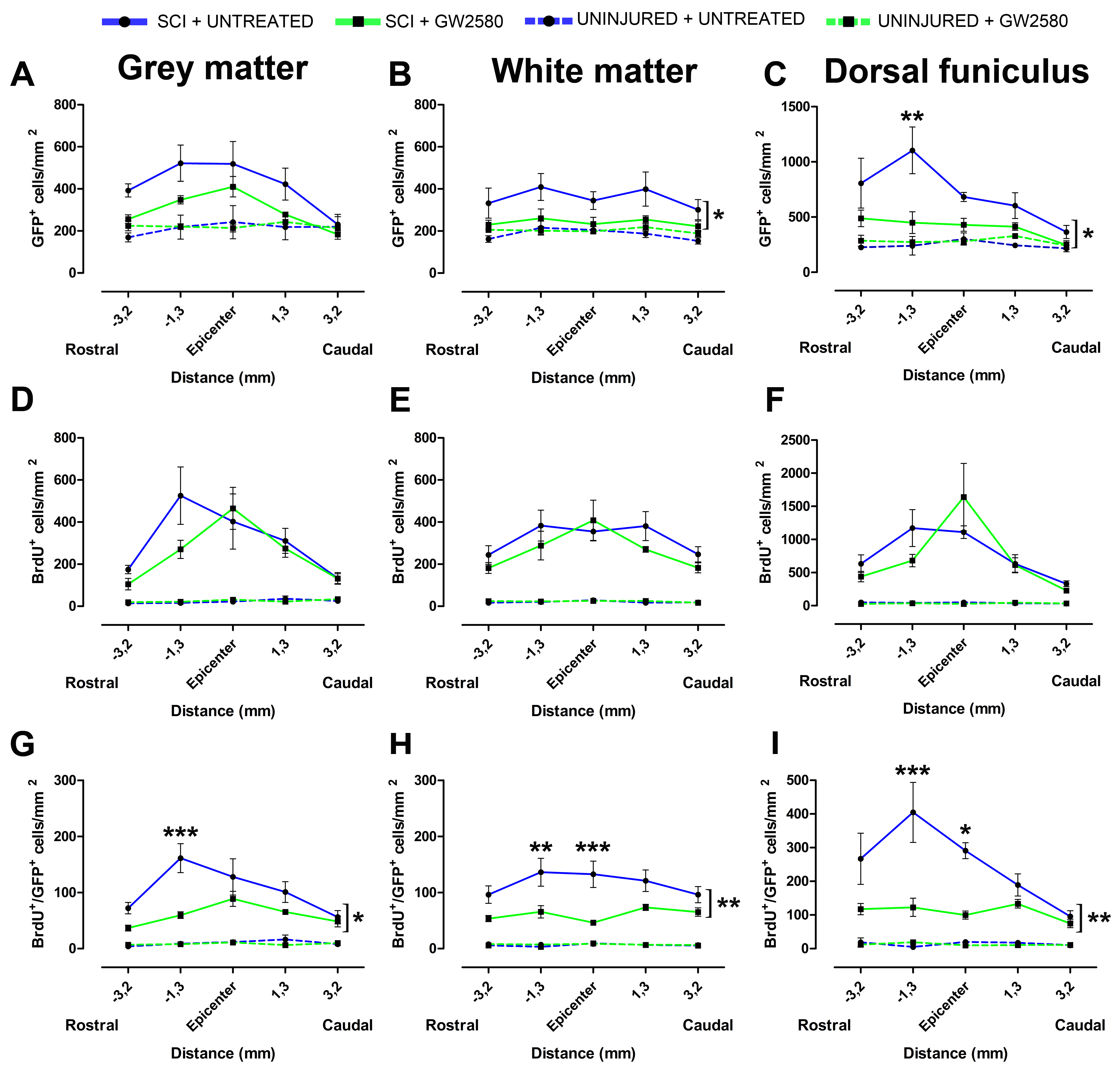

Supplement: FIGURE S1 — Microglia proliferation in GW2580-treated and untreated mice: cell distribution in the spinal cord. Densities of eGFP-positive cells (microglia; A–C), BrdU-positive cells (proliferative cells; D–F) and BrdU/eGFP-positive cells (proliferative microglia; G–I) from the spinal cord of untreated and GW2580-treated mice. Quantifications were done at the lesion epicenter as well as at 3.2 and 1.3 mm distances rostro-caudal to the lesion epicenter in the preserved gray matter (A,D,G), white matter (B,E,H) and in the dorsal funiculus (C,F,I) in untreated and GW2580-treated groups. Uninjured untreated and uninjured GW2580-treated mice are represented as dashed line in all graphs. Data are expressed as number of cells per square millimeters. Data are mean ± SEM per section per group. Two-way ANOVA (*p < 0.05; **p < 0.01) with a post hoc Bonferroni multiple comparison test to compare injured groups (treated and untreated) **p < 0.01; ***p < 0.001. n = 5 for each group. [file Image_1.TIF]

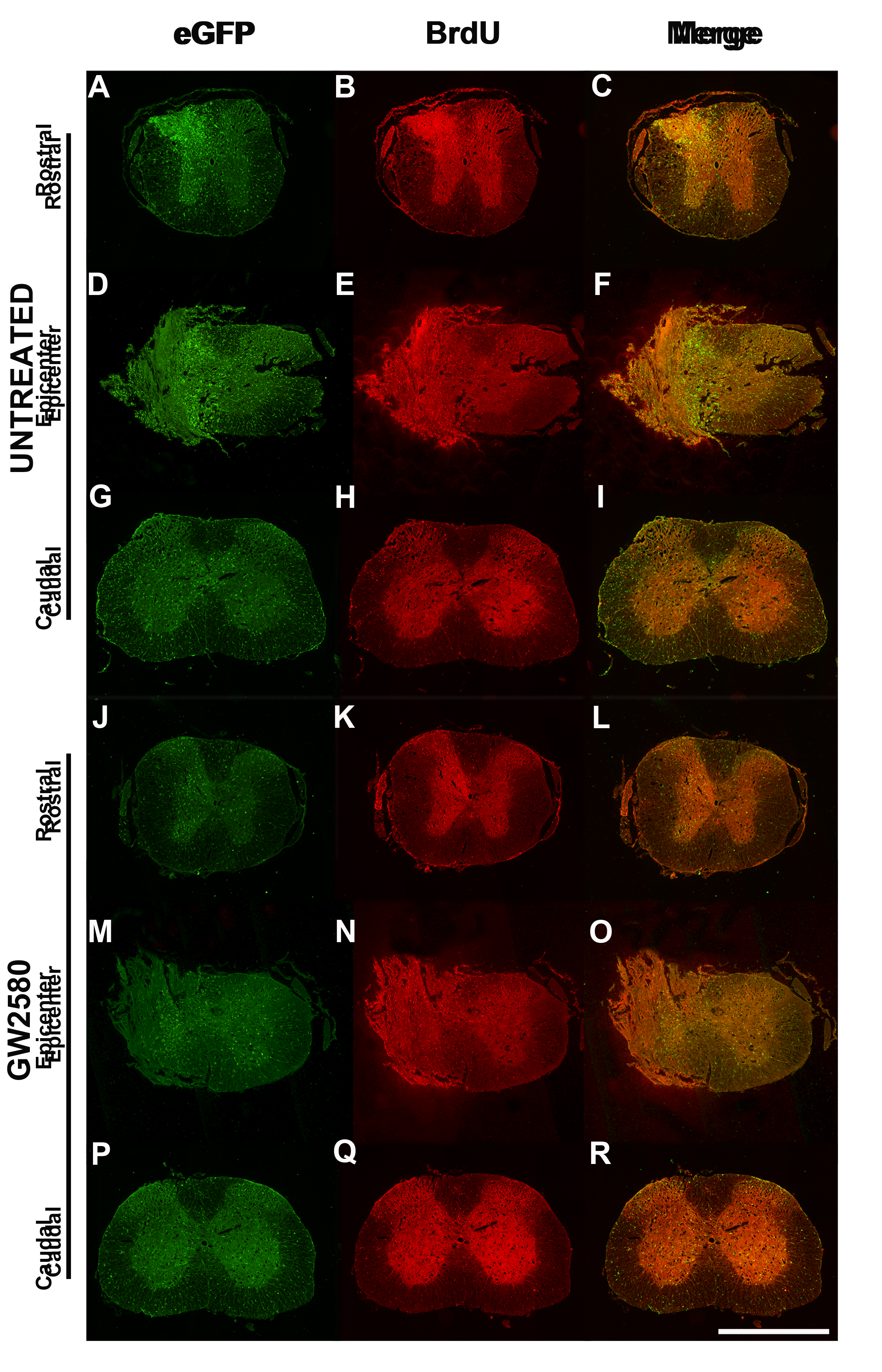

Supplement: FIGURE S2 — GW2580 inhibits microglia proliferation in the mouse spinal cord at 2 weeks after SCI. Fluorescent micrographs of axial spinal cord sections from CX3CR1+/eGFP mice. Microglia eGFP-positive cells (A,D,G,J,M,P), BrdU staining (B,E,H,K,N,Q) and merged (C,F,I,L,O,R). Axial sections from untreated (A–I) and GW2580-treated mice (J–R) at 2 weeks after SCI. Axial images from untreated mice rostral to the lesion epicenter (A–C), at the lesion epicenter (D–F) and caudal to the lesion epicenter (G–I). Axial images from GW2580-treated mice rostral to the lesion epicenter (J–L), at the lesion epicenter (M–O) and caudal to the lesion epicenter (P–R). In all images, the lesion is located on the left side of the spinal cord. Note: displayed rostral and caudal images are each located at 2 mm from the epicenter. Scale bar = 1 mm. [file Image_2.TIF]

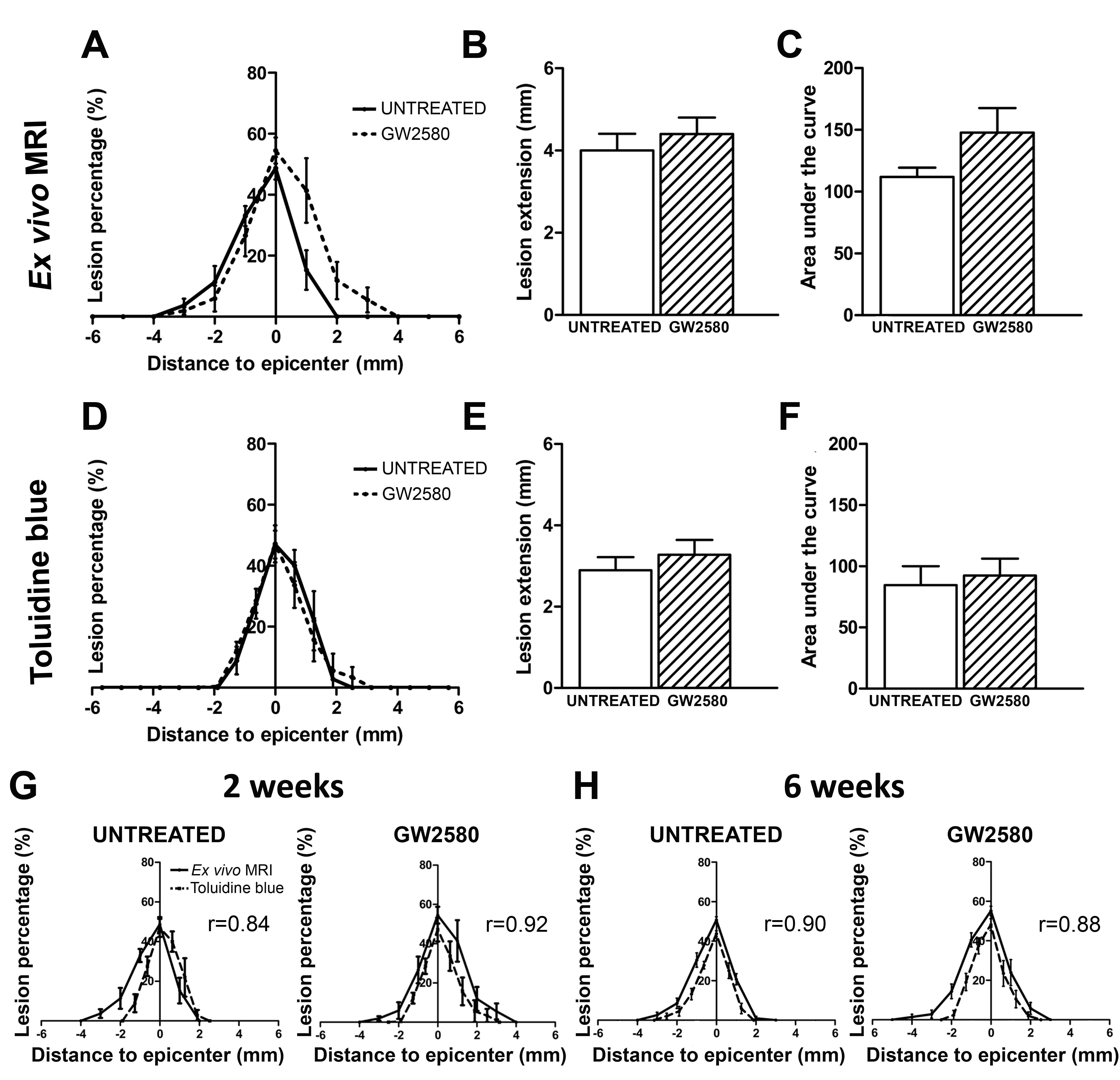

Supplement: FIGURE S3 — Ex vivo MRI and histological assessments of the lesion size in untreated and GW2580-treated mice at 2 weeks after SCI. Ex vivo axial T2-weighted MRI quantification of the lesion area, lesion extension and volume in untreated and GW2580-treated groups (A–C). Toluidine Blue stained axial sections quantification of the lesion area, lesion extension and volume in untreated and GW2580-treated groups (D–F). Correlation between ex vivo and Toluidine Blue analyses in untreated and GW2580 groups at 2 (G) and 6 weeks (H) after SCI. Data are expressed as mean ± SEM per group and per time-point. Student’s unpaired t-test: ns. n = 5 for each group. [file Image_3.TIF]

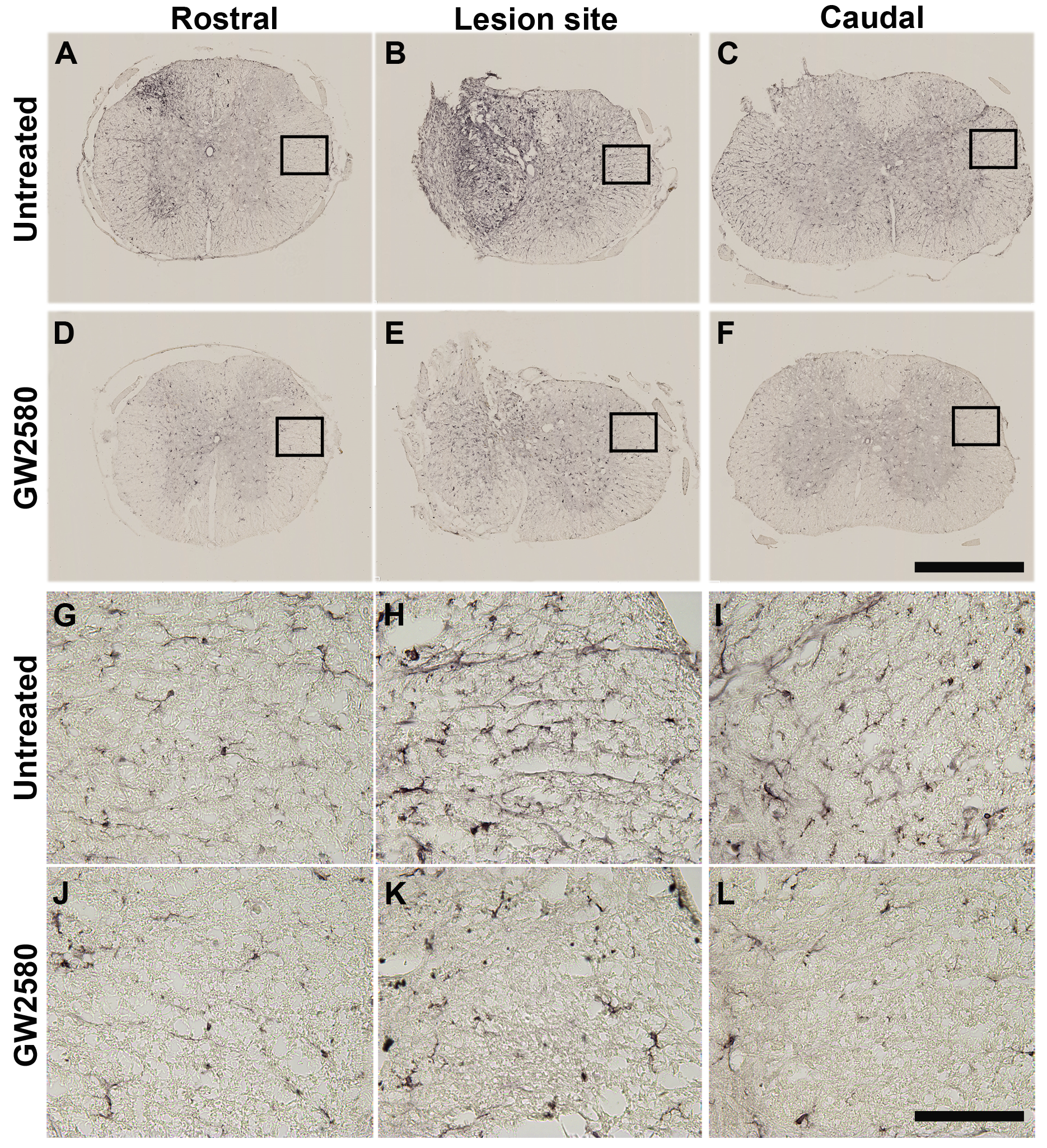

Supplement: FIGURE S4 — IBA1 immunostaining in untreated and GW2580-treated mice at 2 weeks after SCI. Brightfield micrographs representing IBA1 immunostainings rostral (A,D,G,J), within (B,E,H,K) and caudal (C,F,I,L) to the lesion epicenter in untreated mice (A–C,G–I) and in GW2580-treated mice (D–F,J–L) at 2 weeks after SCI. Higher magnification in untreated (G–I) and GW2580-treated mice (J–L) are corresponding to black insets in (A–F). In all images, the lesion is on the left side of the spinal cord. Note: displayed rostral and caudal images are each located at 2 mm from the epicenter. Scale bar = 1 mm (A–F); 100 μm (G–L). [file Image_4.TIF]

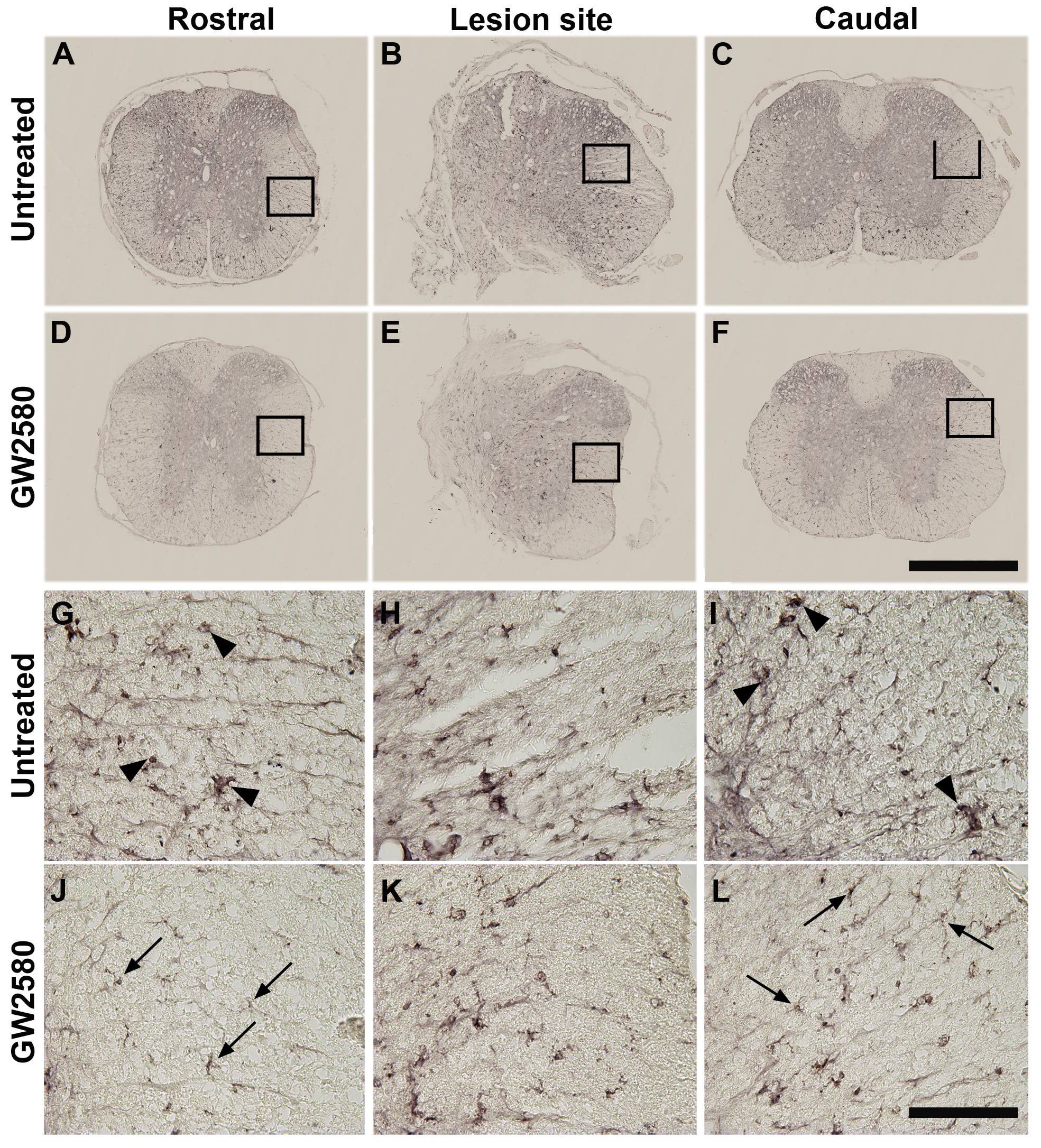

Supplement: FIGURE S5 — IBA1 immunostaining in untreated and GW2580-treated mice at 6 weeks after SCI. IBA1 immunostainings rostral (A,D,G,J), within (B,E,H,K) and caudal (C,F,I,L) to the lesion epicenter in untreated mice (A–C,G–I) and in GW2580-treated mice (D–F,J–L) at 6 weeks after SCI. Higher magnification in untreated (G–I) and GW2580-treated mice (J–L) are corresponding to black insets in (A–F). In all images, the lesion is on the left side of the spinal cord. Note: displayed rostral and caudal images are each located at 2 mm from the epicenter. Scale bar = 1 mm (A–F); 100 μm (G–L). [file Image_5.TIF]

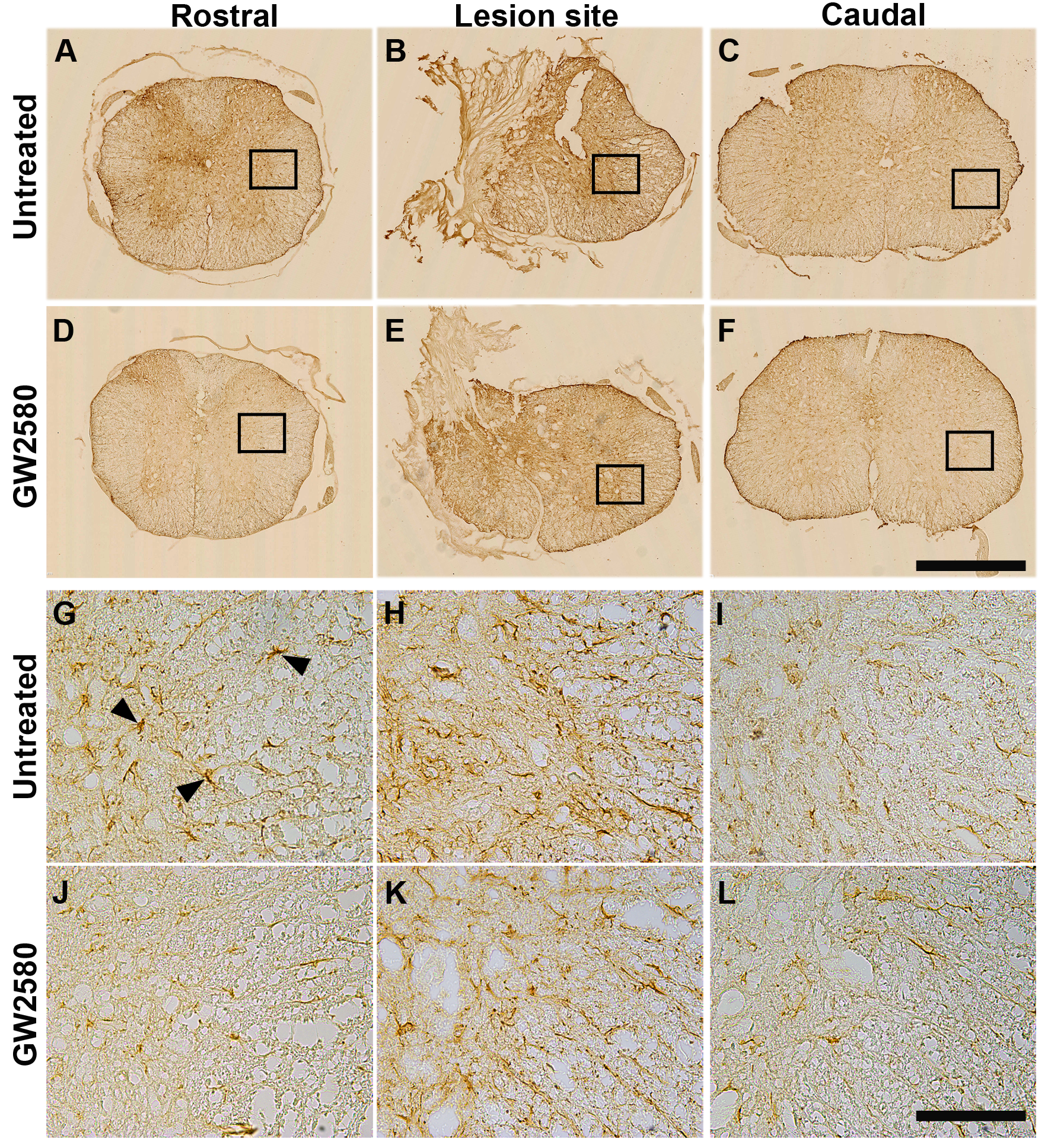

Supplement: FIGURE S6 — GFAP immunostaining in untreated and GW2580-treated mice at 2 weeks after SCI. Brightfield micrographs representing GFAP immunostainings rostral (A,D,G,J), within (B,E,H,K) and caudal (C,F,I,L) to the lesion epicenter in untreated (A–C,G–I) mice and in GW2580-treated (D–F,J–L) mice at 2 weeks after SCI. Higher magnification in untreated (G–I) and GW2580-treated mice (J–L) are corresponding to black insets in (A–F). In all images, the lesion is on the left side of the spinal cord. Note: displayed rostral and caudal images are each located at 2 mm from the epicenter. Scale bar = 1 mm (A–F); 100 μm (G–L). [file Image_6.TIF]

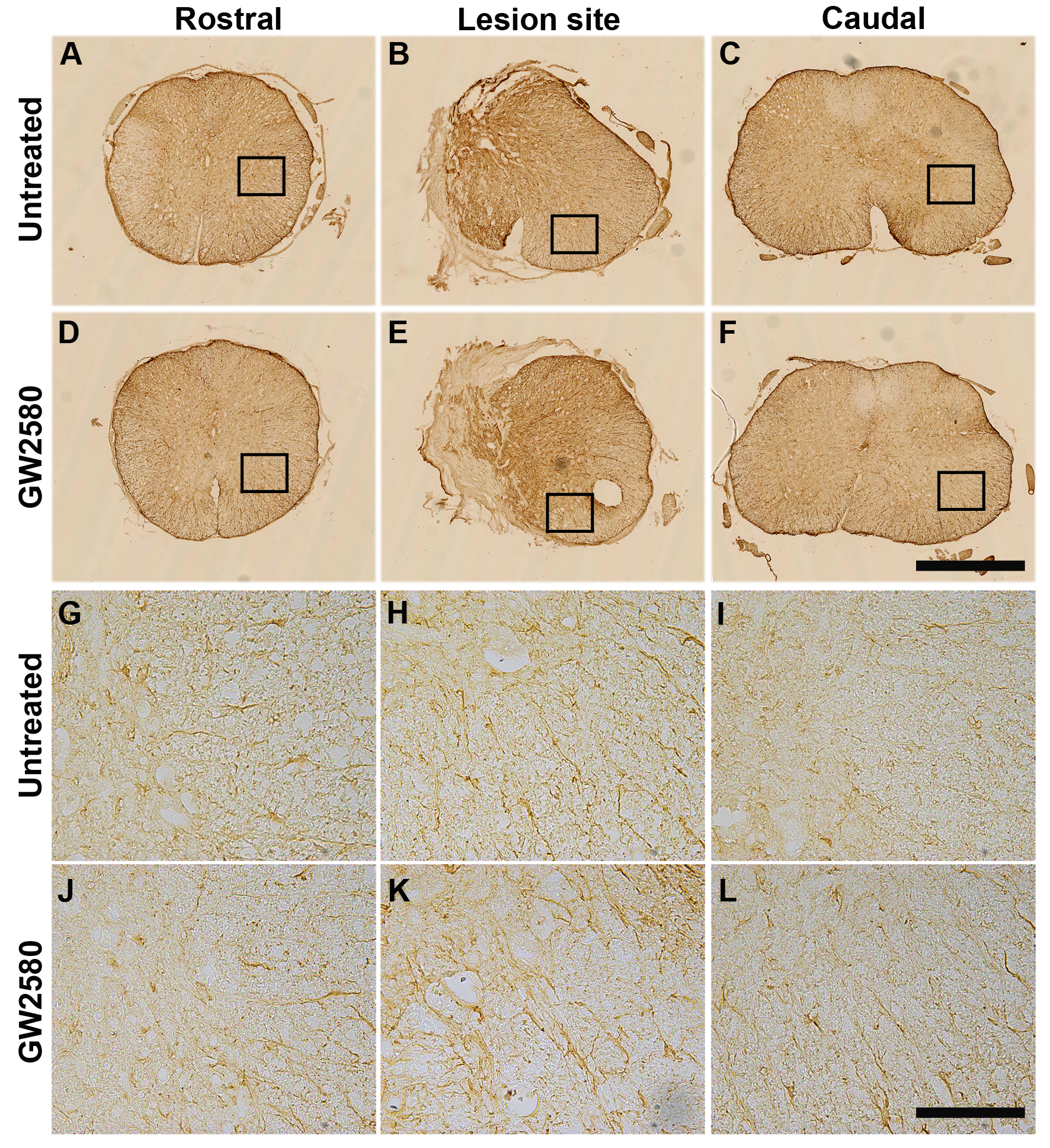

Supplement: FIGURE S7 — GFAP immunostaining in untreated and GW2580-treated mice at 6 weeks after SCI. GFAP immunostainings rostral (A,D,G,J), within (B,E,H,K) and caudal (C,F,I,L) to the lesion epicenter in untreated (A–C,G–I) mice and in GW2580-treated (D–F,J–L) mice at 6 weeks after SCI. Higher magnification in untreated (G–I) and GW2580-treated mice (J–L) are corresponding to black insets in (A–F). In all images, the lesion is on the left side of the spinal cord. Note: displayed rostral and caudal images are each located at 2 mm from the epicenter. Scale bar = 1 mm (A–F); 100 μm (G–L). [file Image_7.TIF]
